# Synthesis of an azido-tagged low affinity ratiometric calcium sensor

**DOI:** 10.1016/j.tet.2015.10.052

**Published:** 2015-12-23

**Authors:** Stuart T. Caldwell, Andrew G. Cairns, Marnie Olson, Susan Chalmers, Mairi Sandison, William Mullen, John G. McCarron, Richard C. Hartley

**Affiliations:** a WestCHEM School of Chemistry, University of Glasgow, Glasgow G12 8QQ, UK; b Strathclyde Institute of Pharmacy and Biomedical Sciences, University of Strathclyde, 161 Cathedral Street, Glasgow G4 0RE, UK; c BHF Glasgow Cardiovascular Research Centre, Institute of Cardiovascular and Medical Sciences, College of Medical Veterinary and Life Sciences, University of Glasgow, Glasgow G12 8QQ, UK

**Keywords:** Calcium, Thiohydantoin, BAPTA, Fluorescence, Bioorthogonal

## Abstract

Changes in high localised concentrations of Ca^2+^ ions are fundamental to cell signalling. The synthesis of a dual excitation, ratiometric calcium ion sensor with a *K*_d_ of 90 μM, is described. It is tagged with an azido group for bioconjugation, and absorbs in the blue/green and emits in the red region of the visible spectrum with a large Stokes shift. The binding modulating nitro group is introduced to the BAPTA core prior to construction of a benzofuran-2-yl carboxaldehyde by an allylation–oxidation–cyclisation sequence, which is followed by condensation with an azido-tagged thiohydantoin. The thiohydantoin unit has to be protected with an acetoxymethyl (AM) caging group to allow CuAAC click reaction and incorporation of the KDEL peptide endoplasmic reticulum (ER) retention sequence.

## Graphical abstract

**Figure:**
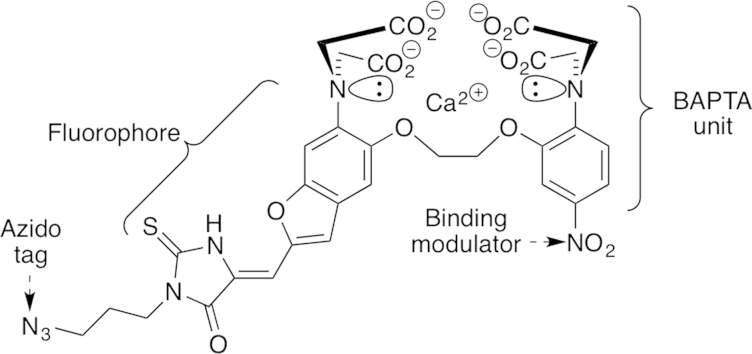

## 1 Introduction

Free Ca^2+^ is an important cell messenger involved in numerous processes such as muscle contraction, cell division and cell death.^1^ The effects of changing the Ca^2+^ concentration, [Ca^2+^], can have a wide reach, extending both within and among cells.^1^ [Ca^2+^] can also affect a range of different activities by acting selectively as a highly localised signal operating in subcellular regions. The free cytosolic [Ca^2+^] is typically in the region of 100 nM at rest and increases to an averaged peak cellular value of <1 μM when the cell is activated.^2^ However, to selectively perform multiple functions, cells localise signals to certain regions by creating high local [Ca^2+^] in the range of tens to several hundred micromolar.^3^ These localised concentrations of Ca^2+^ enable the control of specific cellular activities, such as ion channel and transcription factor activation. In addition to high local concentrations in the cytoplasm, organelles such as the mitochondria, sarcoplasmic reticulum, endoplasmic reticulum and Golgi apparatus each store Ca^2+^ at concentrations (hundreds of micromolar) far above the cytoplasmic average.^4^ Understanding of how [Ca^2+^] selectively controls cell function remains preliminary because of the difficulties in studying Ca^2+^ signals in specific cell regions. Therefore, there have been efforts to develop sensors to study localised and dynamic calcium concentrations.^5^, ^6^, ^7^

As part of these efforts, we set out to develop a fluorescent sensor that would detect changes in the high [Ca^2+^] found in sub-cellular stores. We wanted a sensor that absorbed and emitted in the visible rather than UV region of the spectrum so that its use would disturb the system minimally, and that was ratiometric so that quantification was straightforward. Above all, we wanted to incorporate a tag so that bioorthogonal chemistry could be used to attach a targeting group or to attach the sensor to biomolecules.

We chose to develop a sensor using the highly selective octadentate binding of the Ca^2+^ ion by a 1s,2-bis(*ortho*-aminophenoxy)ethane-*N*,*N*,*N′*,*N'*-tetraacetic acid (BAPTA) ligand,^8^, ^9^ which has become a powerful tool for studying changes in [Ca^2+^].^10^, ^11^, ^12^, ^13^, ^14^, ^15^, ^16^, ^17^, ^18^ Recent work has focused on developing sensors that absorb and emit in the low-energy red and near infrared regions of the spectrum,^14^, ^15^, ^16^, ^17^ both to limit damage and to allow the use of several different fluorophores in the same sample (multiplexing).^18^ In line with this, we decided that our BAPTA-based sensor should absorb and emit in the red region of the spectrum.

We considered an ideal Ca^2+^ sensor would be fluorescent both with Ca^2+^ bound and in its unbound state, but with different absorption or emission wavelengths. This would allow the [Ca^2+^] to be determined directly using the ratio of the two forms using the binding dissociation constant of the sensor (*K*_d_). The use of such so-called ratiometric probes offers many advantages over traditional off/on probes such as correction for artefacts e.g., photobleaching and variation in probe loading.^19^ However, the development of ratiometric Ca^2+^ sensors has lagged behind the development of the traditional off/on probes. Indeed, most biological studies using this technique^20^, ^21^ rely on the original two sensors, Fura-2 and Indo-1,^8^, ^9^ which both absorb in the UV region of the spectrum. There are recent promising ratiometric probes developed by Liu et al.,^13^ but the area is underdeveloped. On the other hand, we and a few others have used Fura-Red^22^, ^23^ as a ratiometric Ca^2+^ sensor that is excited by visible light. It is a dual excitation ratiometric probe so that [Ca^2+^] can easily be determined from the ratio of the emissions at 640 nm when excited at 436 nm (Ca^2+^-bound sensor) and when excited at 472 nm (free sensor).^23^ Fura-Red's very large Stokes shift of ∼200 nm also allows the simultaneous use of other dyes such as Fluo-4.^24^

**Fig. 1:**
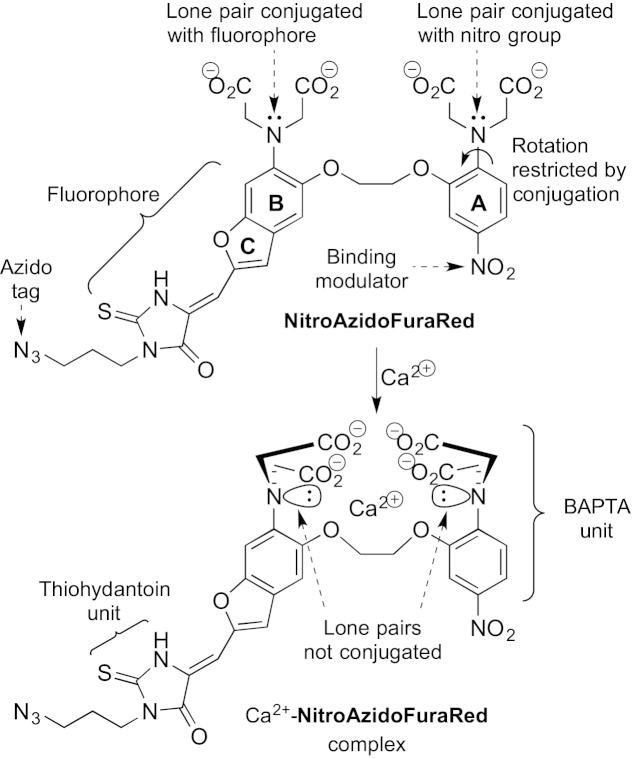
The design of NitroAzidoFuraRed.

Given these excellent properties of Fura-Red, we decided to adjust its binding affinity so that it could detect changes when [Ca^2+^] is high. The binding dissociation constant (*K*_d_) should be midway between the starting and final [Ca^2+^] in the process under study to maximise the observable response of the sensor. Most of the commonly-used Ca^2+^ sensors have *K*_d_ values within the nanomolar range (e.g., Fura-Red has a *K*_d_=380 nM^22^, ^25^), which makes them suitable for measuring global cytosolic [Ca^2+^] fluxes.^10^, ^11^ We reasoned that a low affinity Fura-Red derivative, NitroAzidoFuraRed (Fig. 1), could be prepared that would have an azido tag for bioorthogonal chemistry.^26^

Fura-Red's fluorescent properties arise from photoinduced charge transfer because the fluorophore is in direct conjugation with the BAPTA unit and that conjugation changes when the nitrogen lone pair turns out of plane during binding.^18^ We reasoned that the binding affinity would be decreased by incorporating an electron-withdrawing group on the A ring, which would increase the conjugation with the amino group.^18^ This in turn would disfavour the rotation out of plane that is required for binding to Ca^2+^ and so lower the binding affinity, raising *K*_d_.^11^ We wished to generate a ratiometric probe with a *K*_d_ in the 50–100 μM range that could be used to investigate intra-organellar [Ca^2+^]. Having considered the binding affinities of known Ca^2+^ sensors^10^, ^11^, ^27^ and correlations with Hammett σ constants,^28^ we decided to incorporate a nitro group in the BAPTA core.^29^

We also decided to incorporate an azido tag in NitroAzidoFuraRed because it would offer a universal site of attachment by bioorthogonal chemistry^26^ that could be used to incorporate a targeting group or biomolecule using copper-catalysed or strain-promoted azide–alkyne cycloaddition (CuAAC^16^, ^27^, ^30^ or SPAAC^31^). The azido group would be attached through the thiohydantoin unit so that it would be distal from the BAPTA binding site to minimise potential interference with Ca^2+^ binding.^16^, ^27^, ^30^, ^31^

In summary, we had designed NitroAzidoFuraRed to be a low-affinity, ratiometric, Ca^2+^ sensor excitable with visible light and displaying a large Stokes shift, which would have the potential for attachment to targeting groups and biomolecules. Herein, we show how it was synthesised and present its calcium ion binding properties. We also provide the first examples of CuAAC on thiohydantoin derivatives.

## 2 Results and discussion

There have been no published syntheses of low affinity Fura-Red indicators. Our approach was to construct the BAPTA core **8** by adapting the route of Grynkiewicz et al.^9^ Starting from hydroquinone **1**, benzyl protection gave bisether **2**, which was then converted into the nitro derivative **3**. Selective deprotection of the *ortho*-benzyl group was achieved using aluminium trichloride instead of TFA to give phenol **4** in good yield.^32^ Coupling phenol **4** with known bromide **6**, prepared from phenol **5**,^33^ gave bis-nitro compound **7** in quantitative yield. Reduction of the nitro groups in the presence of the benzyl ether was achieved using iron rather than Pt/H_2_. The use of acetone as a co-solvent was necessary for solubility and critical to the success of this reaction. The resulting diamine was then alkylated to give the tetraethyl ester **8** in excellent yield.

**Scheme 1:**
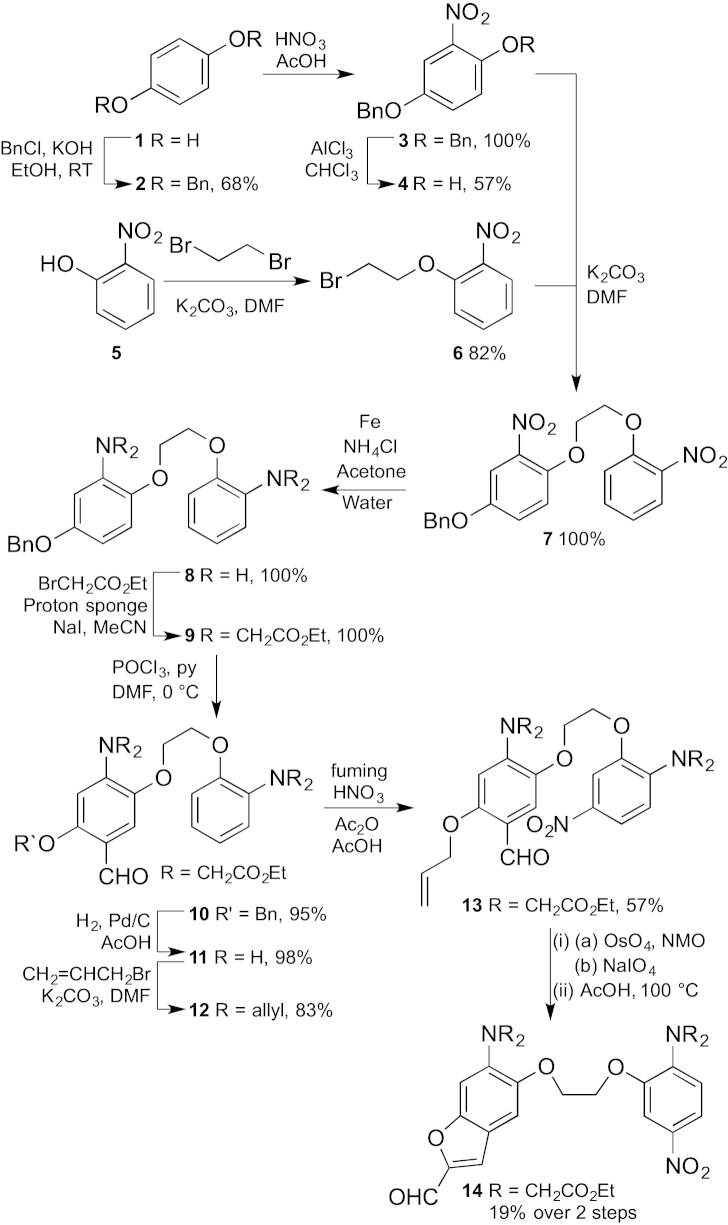

Selective formylation of the more electron rich ring gave aldehyde **10**, which was deprotected by hydrogenolysis to yield phenol **11**. Construction of the benzofuran unit by alkylation with the diethyl acetal of bromoacetaldehyde followed by acid-induced cyclisation^34^ proved capricious, so we decided to investigate allylation of the phenol, followed by oxidative cleavage and cyclisation. This route to benzofuran-2-ylcarboxaldehydes is new and there was only one literature example^35^ of an allyl group being cleaved in the presence of a tertiary amine. The literature example employed ozonolysis, but the use of osmium tetroxide/sodium periodate followed by acid proved satisfactory. Nitration of the A ring has to be carried out after construction of the BAPTA unit and deactivation of the B ring by the aldehyde to take advantage of the directing effect of the amino group,^14^, ^29^, ^36^ and experimentation showed that it was best performed on the allyl ether **12**. Oxidation of the nitrated compound **13** and cyclisation then gave the desired benzofuran **14** in modest yield over the two steps (Scheme 1).

**Scheme 2:**
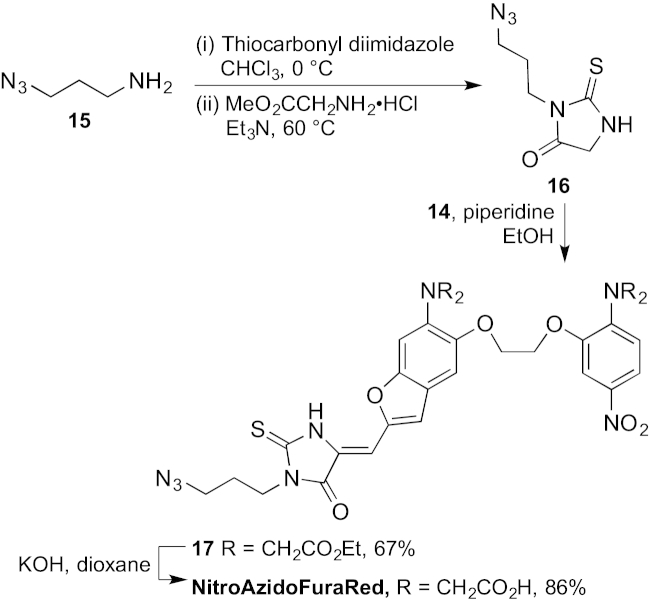

The final part of the synthesis required the incorporation of the thiohydantoin unit (Scheme 2). 3-Azidopropylamine **15** was prepared by the literature method,^37^ converted into an isothiocyanate and reacted with the methyl ester of glycine to give thiohydantoin **16**.^38^ Knoevenagel condensation^39^ with aldehyde **14** completed the fluorophore **17** and saponification of the esters gave NitroAzidoFuraRed.

**Fig. 2:**
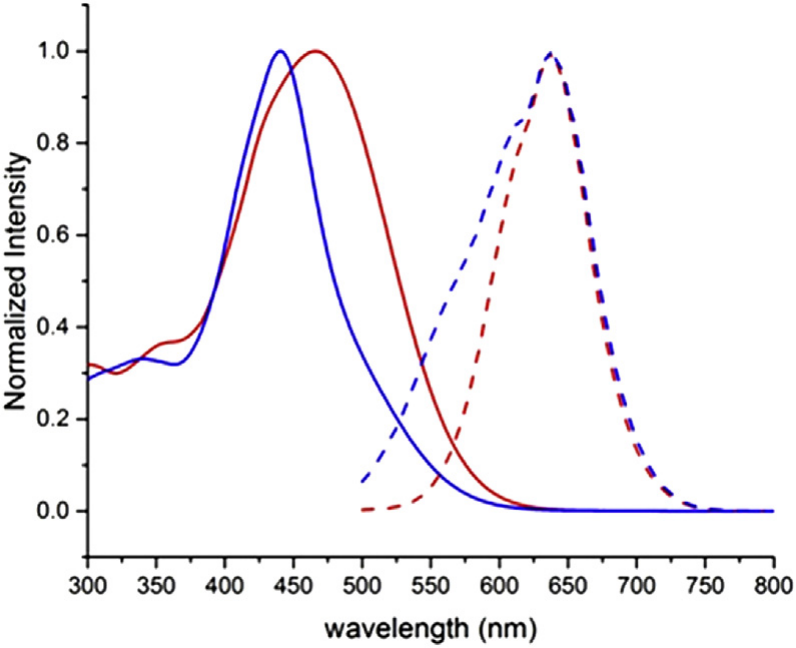
Absorption (solid line) and emission (dashed line) maxima of Ca^2+^-free 43 μM NitroAzidoFuraRed (red) and Ca^2+^ saturated NitroFuraRed (blue) normalised to 1.0. Emission spectra were obtained by excitation at absorption maxima at 465 nm (red) and 440 nm (blue), respectively.

With the desired compound in hand we next investigated the optical properties of the new sensor. As expected, the UV/Vis absorption spectra of the Ca^2+^ free and Ca^2+^ bound NitroAzidoFuraRed are very similar to Fura-Red showing a ∼25 nm blue shift in the absorption maximum upon Ca^2+^ binding (Fig. 2). The fluorescence spectra of the Ca^2+^ free and Ca^2+^ bound NitroAzidoFuraRed are also like Fura-Red showing the expected emission maximum at ∼630 nm.

**Fig. 3:**
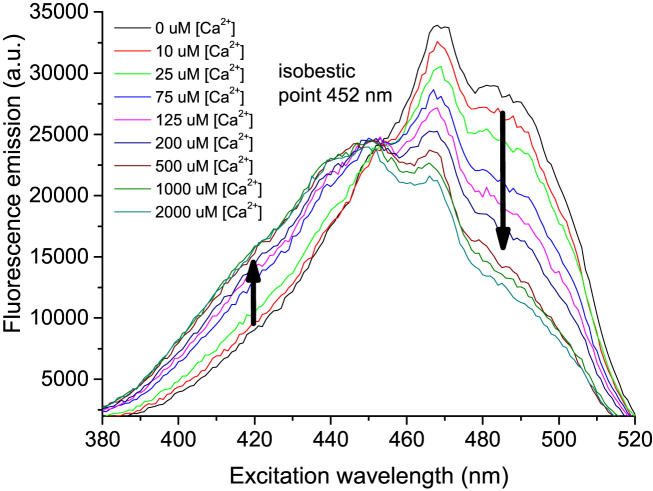
Excitation spectra for NitroAzidoFuraRed with free Ca^2+^ concentrations ranging from 0 μM to 2000 μM. The excitation bandpass was 5 nm and fluorescence emission collected at 630 nm (bandpass 30 nm). The spectra have been corrected for background fluorescence. 5 μM NitroAzido-FuraRed in a 100 mM KCl, 30 mM MOPS, pH 7.2 buffer was incrementally exposed to rising [Ca^2+^] from 0 to 2 mM. With increasing [Ca^2+^], fluorescence emission decreases with excitation above 452 nm and increases with excitation below 452 nm (arrows are drawn at 420 nm and 485 nm).

Having established that the probe possessed the desired optical properties, we turned our attention to the binding properties. The excitation spectra of NitroAzidoFuraRed were obtained with different concentrations of Ca^2+^ and showed a good isobestic point at 452 nm (Fig. 3). The *K*_d_ of the probe was found to be 90 μM using this titration and the ratio of fluorescence intensities at 630 nm when excited at 420 nm and 485 nm, following the method of Grynkiewitz et al.^9^

**Scheme 3:**
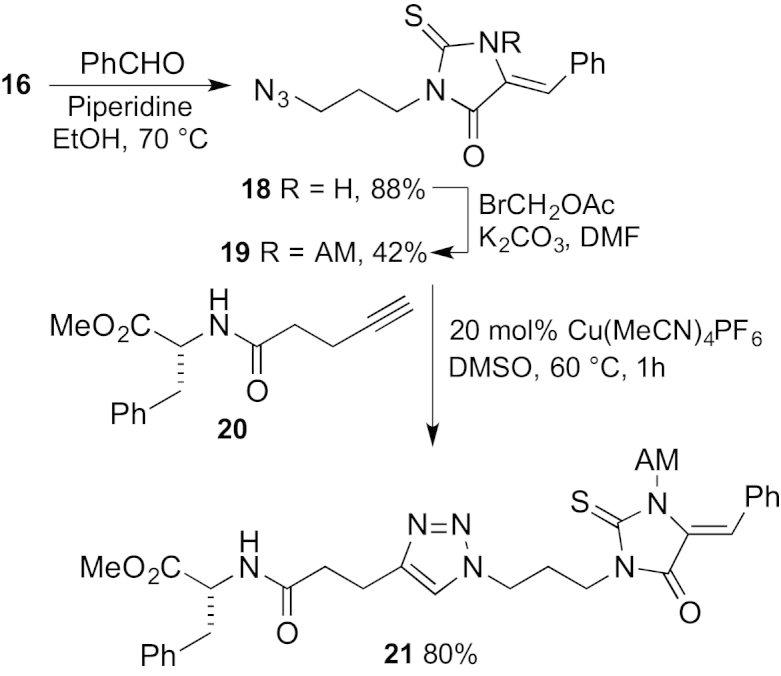

With a sensor with desirable properties in hand, we tested the conjugation reaction. A model thiohydantoin derivative **18**, prepared from thiohydantoin **16**, was used to test the CuAAC with alkyne-tagged phenylalanine derivative **20** (Scheme 3). However, mixtures immediately turned red upon mixing the thiohydantoin with copper(I) ions and the click reaction was ineffective. This is consistent with the reported formation of a thiohydantoin-copper complex.^40^ The tetracarboxylate BAPTA-based fluorophores are cell-impermeable and are generally caged as acetoxymethyl (AM) esters so that they can cross the plasma membrane.^41^ The AM esters are hydrolysed rapidly by esterases inside cells to give the active Ca^2+^ sensors. This strategy has been used for FuraRed,^11^ which also includes *N*-acetylmethyl caging of the thiohydantoin group. Since NitroAzidoFuraRed is designed to be used in cells, we investigated whether AM-protection of the model thiohydantoin would allow CuAAC. Happily, conversion of thiohydantoin **18** into the AM derivative **19** was followed by smooth CuAAC to give the triazole adduct **21**, a process that occurred without the dramatic colour change observed previously.

We then decided to illustrate the conjugation reaction for NitroAzidoFuraRed itself. Proteins to be retained in the endoplasmic reticulum (ER) of cells carry the peptide KDEL motif so that they are recovered from other parts of the cell and returned to the ER.^42^ Inclusion of this retention sequence has been used as a way of localising compounds and nanoparticles in the ER.^43^ Therefore, we exemplified the conjugation of NitroAzidoFuraRed by AM protection followed by CuAAC with an FFKDEL peptide functionalised at the *N-*terminus as the amide of 4-pentynoic acid. Under the conditions tested above, this gave the penta-AM protected triazole in 42% yield.

## 3 Conclusion

In conclusion, we have prepared a Ca^2+^ sensor that is ratiometric, absorbs and emits at long wavelength, has a large Stokes shift, is tuned to low affinity for detection of changes in [Ca^2+^] in intracellular stores and has an azido tag for conjugation to allow localisation in cells. Nitration to introduce the binding-modulator had to be carried out after the synthesis of the BAPTA unit and the introduction of an electron-withdrawing aldehyde, but before introducing the benzofuran moiety. The benzofuran was then constructed using a novel allylation–oxidation–cyclisation. Finally, the azido-tagged thiohydantoin was introduced by Knoevenagel reaction. We then demonstrated the first CuAAC of a thiohydantoin derivative, showing that AM protection is critical to its success, and demonstrated its potential in bioconjugation by attaching an FFKDEL peptide, which incorporates the ER protein retention sequence.

## 4 Experimental section

### 4.1 General

All reactions under an inert atmosphere were carried out using oven-dried or flame dried glassware. Solutions were added via syringe. Dichloromethane and acetonitrile were dried where necessary using a solvent drying system, Puresolv TM, in which solvent is pushed from its storage container under low nitrogen pressure through two stainless steel columns containing activated alumina and copper. Reagents were obtained from commercial suppliers and used without further purification unless otherwise stated. Flash column chromatography was carried out using Fisher matrix silica 60 or using a Biotage Isolera one automated system. ^1^H and ^13^C NMR spectra were obtained on a Bruker AVIII/500 spectrometer operating at 500 and 125 MHz, respectively or a Bruker AVIII/400 spectrometer operating at 400 and 100 MHz, respectively. All coupling constants are measured in Hz. DEPT was used to assign the signals in the ^13^C NMR spectra as C, CH, CH_2_ or CH_3_. EI and CI mass spectra were obtained using the (M Station) JEOL JMS-700 spectrometer. ESI spectra were collected on a Bruker MicroTOF-Q. Infra-red (IR) spectra were obtained on a Shimadzu FTIR-8400S spectrometer using attenuated total reflectance (ATR) so that the IR spectrum of the compound (solid or liquid) could be directly detected (thin layer) without any sample preparation.

### 4.2 Experimental procedures

#### 4.2.1 1,4-Bis(benzyloxy)benzene **2**^31^

1,4-Hydroquinol **1** (40.0 g, 363 mmol, 1.00 equiv) and benzyl chloride (101 ml, 878 mmol, 2.42 equiv) were suspended in ethanol (268 ml). Ethanolic KOH (250 ml, 2.85 M) was added and the mixture stirred mechanically for 3 h under argon then allowed to stand for 3 days. The mixture was quenched in H_2_O and the off-white precipitate was filtered and crystallised in eight portions (14 g/500 ml) from boiling EtOH. The combined crystallisation leftovers were combined, then concentrated to give another crop of crystals. The combined solid was dried under vacuum to give ether **2** as needles (71.8 g, 68%). *δ*_H_ (400 MHz, CDCl_3_): 7.45–7.28 (10H, m, OCH_2_*Ph*), 6.90 (4H, s, *Ph*(OBn)_2_), 5.01 (4H, s, O*CH*_2_Ph). ^13^C NMR (101 MHz, CDCl_3_) *δ* 153.26 (C), 137.37 (C), 128.68 (CH), 128.02 (CH), 127.60 (CH), 115.90 (CH), 70.74 (CH_2_). Spectroscopic data agree with lit.^31^

#### 4.2.2 1,4-Bis(benzyloxy)-2-nitrobenzene **3**^31^

Nitric acid (70%) (19.5 ml, 437.4 mmol, 2.0 equiv) was added slowly to a suspension of hydroquinol **2** (63.5 g, 218.7 mmol, 1.0 equiv) in glacial acetic acid (400 ml) at 0 °C. The solution was stirred at 0 °C for 3 h, poured into water (∼1500 ml) and the resulting precipitate filtered off. The precipitate was washed with water (1500 ml), dissolved in CHCl_3_ (300 ml), dried over magnesium sulfate and concentrated under vacuum to give nitro compound **3** as a yellow solid. (73.04 g, 100%). mp 80–81 °C (lit.: 80–81 °C^44^). *δ*_H_ (400 MHz, CDCl_3_): 7.49 (1H, d, *J*=3.0, H3), 7.46–7.30 (10H, m, OCH_2_*Ph*), 7.12 (1H, dd, *J*=9.2, 3.1 Hz, H5), 7.05 (1H, d, *J*=9.2 Hz, H6), 5.18 (2H, s, CH_2_), 5.05 (2H, s, CH_2_). ^13^C NMR (101 MHz, CDCl_3_) *δ* 152.30 (C), 146.39 (C), 135.97 (C), 135.88 (C), 128.75 (CH), 128.70 (CH), 128.37 (CH), 128.22 (CH), 127.59 (CH), 127.15 (CH), 121.54 (CH), 117.24, 111.18 (CH), 72.10 (CH_2_), 70.94 (CH_2_). IR (ATR cm^−1^): 1520 (NO_2_), 1497 (C_Ar_=C_Ar_), 1341 (NO_2_). MS (ESI^+^): 358 [(M+Na)^+^, 100]. HRMS: 358.1034. C_20_H_17_NNaO_4_ requires 358.1050. Spectroscopic data agree with lit.^31^

#### 4.2.3 4-Benzyloxy-2-nitrophenol **4**^31^

Nitroaryl **3** (4.50 g, 13.4 mmol, 1.00 equiv) was dissolved in CHCl_3_ (65 ml) and stirred at 0 °C under argon. To the mixture a suspension of AlCl_3_ (2.07 g, 15.5 mmol, 1.16 equiv) in CHCl_3_ (10 ml) was added portionwise with additional CHCl_3_ (20 ml). The reaction was monitored by TLC and was complete after 15 min. The mixture was quenched into HCl_(aq)_ (1 M) and extracted with DCM (× 3). The combined organics were dried (MgSO_4_), filtered and the solvent removed under reduced pressure. The residue was crystallised from boiling MeOH, then recrystallised from boiling MeOH with 5% H_2_O added. The resulting crystals were washed (H_2_O), then partitioned between H_2_O and DCM. The organic layer was separated and the solvent removed under reduced pressure, giving nitrophenol **4** as a solid (1.88 g, 57%). *δ*_H_ (400 MHz, CDCl_3_): 10.36 (1H, s, ArOH), 7.61 (1H, d, *J*=3.1 Hz, H3), 7.46–7.33 (5H, m, OCH_2_Ph), 7.29 (1H, dd, *J*=9.2, 3.1 Hz, H5), 7.10 (1H, d, *J*=9.2 Hz, H6), 5.06 (2H, s, PhCH_2_). ^13^C NMR (101 MHz, CDCl_3_) *δ* 151.64 (C), 150.20 (C), 135.86 (C), 132.97 (C), 128.76 (CH), 128.42 (CH), 127.88 (CH), 127.65 (CH), 120.93 (CH), 107.19 (CH), 70.92 (CH_2_). IR υ_max_ (ATR)/cm^−1^: 3231 (O–H), 3123 (C_Ar_-H), 3106 (C_Ar_-H), 1584 (C_Ar_=C_Ar_), 1530 (NO_2_), 1501 (C_Ar_=C_Ar_), 1485 (C_Ar_=C_Ar_), 1316 (NO_2_). MS (EI^+^): 245 (M^+^, 18%), 91 (C_7_H_7_^+^, 100). HRMS: 245.0696. C_13_H_11_O_4_N requires 245.0688. Spectroscopic data agree with lit.^31^

#### 4.2.4 1-(2-Bromoethoxy)-2-nitrobenzene **6**^33^

2-Nitrophenol **5** (3.99 g, 28.7 mmol, 1.00 equiv), 1,2-dibromoethane (7.5 ml, 87 mmol, 3.0 equiv) and K_2_CO_3_ (4.36 g, 31.6 mmol, 1.10 equiv) were combined in DMF (6.0 ml) and stirred at 120 °C for 3 h under argon. The precipitate was filtered off and washed with DCM and H_2_O. The filtrate layers were separated and the organics were washed with 0.5 M NaOH_(aq)_ and saturated brine solution. The organics were dried (MgSO_4_) and filtered, then the solvent was removed under reduced pressure to give the bromide **6** (4.53 g, 65%) as a solid. mp 36–40 °C (lit.: 36–38 °C^45^).*δ*_H_ (400 MHz, CDCl_3_): 7.85 (1H, dd, *J*=8.3, 1.7 Hz, H6), 7.55 (1H, ddd, *J*=8.4, 7.5, 1.7 Hz, H4), 7.12–7.06 (2H, m, H3, H5), 4.42 (2H, t, *J*=6.5 Hz, OC*H*_2_CH_2_Br), 3.68 (2H, t, *J*=6.5 Hz, OCH_2_C*H*_2_Br). Data agree with lit.^33^

#### 4.2.5 2-Nitro-1-[2′-(4″-benzyloxy-2″-nitrophenoxy)ethoxy]benzene **7**

1-(2-Bromoethoxy)-2-nitrobenzene **6** (10.9 g, 44.3 mmol, 1.47 equiv), nitrophenol **4** (7.0 g, 30 mmol, 1.0 equiv) and K_2_CO_3_ (3.0 g, 22 mmol, 0.7 equiv) were combined in dry DMF (17.0 ml) and stirred at 140 °C for 2 h under argon, then allowed to stir at rt for 12 h. The material was heated to 50 °C, and the precipitate was dissolved with dropwise addition of water. After 24 h stirring precipitate had formed and was collected by filtration. The material was then washed out in a mixture of acetone and hexane. The solvent was removed under reduced pressure and the compound dissolved in DCM then washed with water (× three) and 5% LiCl_(aq)_ solution, then the solvent removed from the combined organics to give the ether **7** in quantitative yield (12.4 g, quant). mp 110–111 °C. *δ*_H_ (400 MHz, CDCl_3_): 7.84 (1H, dd, *J*=8.1, 1.7 Hz, H-3), 7.57 (1H, ddd, *J*=8.5, 7.4, 1.7 Hz, H-5), 7.47–7.32 (6H, m, OCH_2_*Ph*, H-3″), 7.23–7.19 (3H, m, H6, H5″, H6″), 7.12–7.05 (1H, m, H4), 5.07 (2H, s, OC*H*_2_Ph), 4.52–4.46 (4H, m, OC*H*_2_C*H*_2_O). *δ*_C_ (CDCl_3_, 100 MHz): 152.95 (C), 151.956 (C), 146.37 (C), 140.63 (C), 140.20 (C), 135.90 (C), 134.34 (CH), 128.76 (CH), 128.38 (CH), 127.59 (CH), 125.68 (CH), 121.73 (CH), 121.24 (CH), 118.75 (CH), 115.61 (CH), 111.04 (CH), 70.96 (CH_2_), 69.99 (CH_2_), 68.77 (CH_2_). υ_max_ (ATR)/cm^−1^: 3094 (CH), 2960 (CH), 2929 (CH), 2871 (CH), 1606 (C_Ar_=C_Ar_), 1518 (NO_2_), 1497 (C_Ar_=C_Ar_), 1487 (NO_2_), 1451 (C_Ar_=C_Ar_). MS (EI^+^): (410 M^+.^, 45%), 166 (O_2_NC_6_H_4_OCH_2_CH_2_^+^, 38), 122 (C_4_H_4_NO_2_^+^, 44), 91 (PhCH_2_^+^, 100). HRMS: 410.1114. C_21_H_18_N_2_O_7_ requires M^+^ 410.1114.

#### 4.2.6 2-[2′-(2″-Aminophenoxy)ethoxy]-5-(benzyloxy)aniline **8**

Iron powder (8.44 g, 151.13 mmol, 7.7 equiv) was added portionwise to a solution of dinitro compound **7** (8.05 g, 19.61 mmol, 1.0 equiv) and ammonium chloride (6.47 g, 121.04 mmol, 6.17 equiv) in acetone: water (300 ml (4:1)). The suspension was stirred rapidly and heated to 70 °C under argon overnight. After cooling to rt the mixture was filtered through Celite eluting with acetone, the solvent was removed under reduced pressure. The residue was dissolved in DCM, filtered through Celite again eluting with dichloromethane. The dichloromethane layer was washed with brine, dried (MgSO_4_), filtered and the solvent removed under reduced pressure to give diamine **8** as a solid (6.23 mg, 91%). mp 146  C. *δ*_H_ (400 MHz, CDCl_3_): 7.44–7.34 (4H, m, H2‴, H3‴, H5‴, H6‴) 7.33–7.27 (1H, m, H4‴), 6.86–6.79 (2H, m, H4″, H6″), 6.77 (1H, d, *J*=8.7 Hz, H3), 6.74–6.68 (2H, m, H3″, H5″), 6.39, (1H, d, *J*=2.9 Hz, H6), 6.30 (1H, dd, *J*=8.7, 2.9 Hz, H5), 4.98 (2H, s, OC*H*_*2*_Ph), 4.35–4.27 (4H, m, OC*H*_*2*_C*H*_*2*_O), 3.83 (4H, s, NH_2_). *δ*_C_ (100 MHz, CDCl_3_): 154.37 (C), 146.26 (C), 140.85 (C), 138.24 (C), 137.47 (C), 136.89 (C), 128.52 (CH), 127.81 (CH), 127.43 (CH), 121.94 (CH), 118.38 (CH), 115.36 (CH), 114.31 (CH), 112.66 (CH), 103.46 (CH), 103.10 (CH), 70.43 (CH_2_), 68.64 (CH_2_), 67.64 (CH_2°_). IR (ATR cm^−1^): 3454 (NH), 3433 (NH), 3367 (NH), 3350 (NH), 3063 (C_Ar_–H), 3038 (C_Ar_–H), 2959 (C–H), 2939 (C–H), 2886 (C–H), 1611 (C_Ar_=C_Ar_), 1601 (C_Ar_=C_Ar_), 1595 (C_Ar_=C_Ar_), 1207 (C–O). MS (EI^+^): 350 (M^+^, 70%), 241 (M^+^–C_6_H_4_NH_2_O, 9), 215 (C_13_H_13_NO_2_^+^, 42), 214 (C_13_H_14_NO_2_^+^, 11), 136 (C_8_H_9_NO^+^, 41), 91 (C_7_H_7_^+^, 100). HRMS: 350.1629 requires 350.1630.

#### 4.2.7 Ethyl 2-*N*-(2″-{2‴-[4‴′-(benzyloxy)-2‴′-*N,N-*(2‴″-ethoxy-2‴″-oxoethyl)amino]phenoxy]ethoxy}phenyl)-*N*-(2′-ethoxy-2′-oxoethyl)amino]acetate **9**

Aniline **8** (1.80 g, 5.14 mmol, 1.00 equiv) was dried by azeotrope twice in PhMe then combined under argon with NaI (2.12 g, 14.1 mmol, 2.75 equiv, dried under vacuum at 100 °C for 6 h) and proton sponge (also dried by azeotrope in PhMe, 7.50 g, 35.0 mmol, 6.81 equiv) dry MeCN (10.0 ml) and ethyl bromoacetate (5.00 ml, 46.3 mmol, 9.01 equiv) were added to the stirring mixture which was then heated under reflux for 24 h. The solvent was then removed under reduced pressure and the residue was dissolved in PhMe and filtered. The filtrate was washed five times with dilute HCl_(aq)_, then the organics were then combined and the solvent removed under vacuum. Crystallisation (Pet. ether/EtOAc) gave the diamine tetraester **9** (4.00 g, quant.) as needles. mp 96–97 °C. *δ*_H_ (400 MHz, CDCl_3_): 7.45–7.34 (4H, m, H2‴″, H3‴″, H5‴″, H6‴″) 7.33–7.28 (1H, m, H4‴″), 6.93–6.78 (4H, m, H3″, H4″, H5″, H6″), 6.76 (1H, d, *J*=9.5 Hz, H6‴′), 6.50–6.44 (2H, m, H3‴′, H5‴′), 4.97 (2H, s, OC*H*_*2*_Ph), 4.23 (4H, s, OC*H*_*2*_C*H*_*2*_O), 4.16 (4H, s, NCH_2_), 4.14 (4H, s, NCH_2_), 4.055 (4H, q, *J*=7.2 Hz, CO_2_C*H*_2_), 4.053 (4H, q, *J*=7.2 Hz, CO_2_CH_2_), 1.16 (6H, t, *J*=7.1 Hz, CH_2_C*H*_3_), 1.15 (6H, t, *J*=7.1 Hz, CH_2_C*H*_3_). *δ*_C_ (100 MHz, CDCl_3_): 171.60 (C), 171.42 (C), 153.61 (C), 150.32 (C), 144.73 (C), 140.58 (C), 139.36 (C), 137.22 (C), 128.53 (CH), 127.89 (CH), 127.53 (CH), 122.13 (CH), 121.40 (CH), 118.96 (CH), 114.43 (CH), 113.09 (CH), 107.22 (CH), 106.37 (CH), 70.45 (CH_2_), 67.81 (CH_2_), 67.11 (CH_2_), 60.80 (CH_2_), 60.75 (CH_2_), 53.49 (CH_2_), 53.44 (CH_2_), 14.06 (CH_3_), 14.03 (CH_3_). IR υ_max_ (ATR)/cm^−1^: 2986 (C_Ar_=C_Ar_), 2932b (C_Ar_=C_Ar_), 1736 (CO), 1598 (C_Ar_=C_Ar_), 1512 (C_Ar_=C_Ar_). MS (NSI): 717 [(M+Na^+^), 52%], 695 [(M+H)^+^, 100]. HRMS: 695.3173. C_37_H_47_O_11_N_2_ requires 695.3180.

#### 4.2.8 Ethyl 2-[(2″-{2‴-[4‴′-(benzyloxy)-2‴′-[bis(2‴″-ethoxy-2‴″-oxoethyl)amino]5-formylphenoxy]ethoxy}phenyl) (2′-ethoxy-2′-oxoethyl)amino]acetate **10**

Phosphorus oxychloride (6.0 ml, 64.94 mmol, 8.0 equiv) was added slowly to a solution of tetraester **9** (5.64 g, 8.11 mmol, 1.00 equiv) and pyridine (6.54 ml, 81.1 mmol, 10.0 equiv) in DMF (40 ml) at 0 °C under argon. The resulting red solution was stirred at 0 °C for 1.5 h then slowly quenched with cold KOH solution (1M). The solution was extracted in dichloromethane (3×70 ml), the combined organic layers were washed with brine (4×200 ml) dried over magnesium sulfate and concentrated under vacuum to give the aldehyde suitably pure to use without further purification (5.60 g, 95%). mp 135–136 °C. *δ*_H_ (400 MHz, CDCl_3_): 10.31 (1H, s, CHO) 7.42–7.31 (5H, m, Ph), 7.29 (1H, s, H6‴′), 6.93–6.79 (4H, m, H3″, H4″, H5″ H6″), 6.30 (1H, s, H3‴′), 5.10 (2H, s, OC*H*_*2*_Ph), 4.27–4.20 (4H, m, OC*H*_*2*_C*H*_*2*_O), 4.18 (4H, s, NCH_2_), 4.14 (4H, s, NCH_2_), 4.06 (4H, q, *J*=7.1 Hz, CO_2_CH_*2*_), 4.03 (4H, q, *J*=7.1 Hz, CO_2_CH_2_), 1.16 (6H, t, *J*=7.2 Hz, CH_2_C*H*_3_), 1.14 (6H, t, *J*=7.1 Hz, CH_2_C*H*_3_). *δ*_C_ (100 MHz, CDCl_3_): 187.49 (CH), 171.51 (C), 170.7051 (C), 157.34 (C), 150.19 (C), 146.44 (C), 143.96 (C), 139.48 (C), 136.32 (C), 128.71 (CH), 128.24 (CH), 127.29 (CH), 122.10 (CH), 121.66 (CH), 119.10 (CH), 117.98 (C), 113.32 (CH), 110.62 (CH), 102.67 (CH), 71.10 (CH_2_), 67.61 (CH_2_), 66.90 (CH_2_), 61.22 (CH_2_), 60.76 (CH_2_), 53.86 (CH_2_), 53.50 (CH_2_), 14.07 (CH_3_), 14.02 (CH_3_). IR (ATR cm^−1^): 2975 (C_Ar_–H), 2938 (C_Ar_–H), 2928 (C_Ar_–H), 2873 (C_Ar_–H), 1745 (CO), 1718 (CO), 1662, 1598 (C_Ar_=C_Ar_), 1517 (C_Ar_=C_Ar_), 1508 (C_Ar_=C_Ar_). MS (NSI): 1462 [(2M+NH_4_^+^), 6%], 761 [(M+K^+^), 3], 745 [(M+Na^+^), 60], 740 [(M+NH_4_^+^), 45], 723 [(M+H^+^), 100]. HRMS: 723.3121 requires 723.3129, C_38_H_47_O_12_N_2_.

#### 4.2.9 Ethyl 2-{[2″-(2‴-{2‴′-[bis(2‴″-ethoxy-2‴″-oxoethyl)amino]-5‴′-formyl-4‴′-hydroxyphenoxy}ethoxy)phenyl](2′-ethoxy-2′-oxoethyl)amino}acetate **11**

Palladium on carbon (10% loading by weight, 600 mg) was added to a solution of aldehyde **10** (2.04 g, 2.82 mmol, 1.00 equiv) in acetic acid (30 ml). The suspension was flushed with H_2(g)_ then stirred under a H_2(g)_ atmosphere overnight. After this time, the solution was filtered through Celite and the Celite washed with EtOAc. The combined organic solvents were concentrated under vacuum. Any residual acetic acid was removed by toluene azeotrope to give phenol **11** as an off white solid. (1.75 g, 98%). mp 78–80 °C. *δ*_H_ (400 MHz, CDCl_3_): 11.21 (1H, s, PhOH), 9.61 (1H, s, CHO), 6.92 (1H, s, H6‴′), 6.94–6.82 (4H, m, H3″, H4″, H5″ H6″), 6.15 (1H, s, H3‴′), 4.23 (4H, s, OCH_2_CH_2_O), 4.22 (4H, s, NCH_2_), 4.15 (4H, s, NCH_2_), 4.08 (4H, q, *J*=7.1 Hz, CO_2_CH_2_), 4.08 (4H, q, *J*=7.1 Hz, CO_2_CH_2_), 1.19 (6H, t, *J*=7.2 Hz, CH_2_C*H*_3_), 1.16 (6H, t, *J*=7.2 Hz, CH_2_C*H*_3_). *δ*_C_ (100 MHz, CDCl_3_): 193.22 (CH), 171.49 (C), 170.37 (C), 158.82 (C), 150.17 (C), 148.32 (C), 142.72 (C), 139.48 (C), 122.25 (CH), 121.78 (CH), 119.23 (CH), 115.90 (CH), 113.38 (CH), 113.06 (C), 104.22 (CH), 68.23 (CH_2_), 66.92 (CH_2_), 61.34 (CH_2_), 60.78 (CH_2_), 53.85 (CH_2_), 53.48 (CH_2_), 14.09 (CH_3_), 13.97 (CH_3_). υ_max_ (ATR)/cm^−1^: 2977 (C_Ar_–H), 2960 (C_Ar_–H), 2937 (C_Ar_–H), 2908 (C_Ar_–H), 2877 (C_Ar_–H), 1739 (CO), 1630 (CO), 1507 (C_Ar_=C_Ar_). MS (NSI): 671 [(M+K^+^), 5%], 655 [(M+Na^+^), 62], 650 [(M+NH_4_^+^), 22], 633 [(M+H^+^), 100]. HRMS: 633.2652. (M+H)^+^ C_31_H_41_O_12_N_2_ requires 633.2659.

#### 4.2.10 Ethyl 2-{[2″-(2‴-{2‴′-[bis(2‴″-ethoxy-2‴″-oxoethyl)amino]-5‴′-formyl-4‴′-(prop-2‴‴-en-1‴‴-yloxy)phenoxy}ethoxy)phenyl](2′-ethoxy-2′-oxoethyl)amino}acetate **12**

Allyl bromide (6.90 ml, 79.52 mmol, 4.0 equiv) was added to a stirred suspension of phenol **11** (12.58 g, 19.88 mmol), K_2_CO_3_ (10.99 g, 79.52 mmol, 4.0 equiv) and KI (3.30 g, 19.88 mmol, 1.0 equiv) in DMF (100 ml). The suspension was heated to 100 °C overnight under an atmosphere of argon. After cooling to rt the suspension was acidified to pH 1 with 1M hydrochloric acid and extracted onto EtOAc (2×150 ml). The combined organic layers were washed with brine (3×300 ml), dried over magnesium sulfate and concentrated under vacuum. The resulting residue was passed though a large plug of silica eluting EtO_2_ then concentrated under vacuum. The residue was then recrystallised from EtO_2_:Hexane to give *allyl ether* **12** as off white solid (11.08 g, 83%). mp 95–96 °C. *δ*_H_ (500 MHz: CDCl_3_): 10.27 (1H, s, CHO), 7.27 (1H, s, H-6), 6.89–6.79 (4H, m, Ar–H), 6.26 (1H, s, H-3), 6.01 (1H, ddd, *J*=17.2, 10.5, 5.2 Hz, C*H*=CH_2_), 5.40 (1H, ddd, *J*=17.3, 3.0, 1.5 Hz, CH=C*H*_*A*_H_B_), 5.31 (1H, dq, *J*=10.5, 1.3 Hz, CH=CH_A_*H*_*B*_), 4.56 (2H, dt, *J*=5.2, 1.5 Hz, OCH_2_), 4.25–4.21 (4H, m, OCH_2_CH_2_O), 4.21 (4H, s, 2×NCH_2_), 4.13 (4H, s, 2×NCH_2_), 4.06 (4H, q, *J*=7.1 Hz, 2×CO_2_CH_2_), 4.04 (4H, q, *J*=7.1 Hz, 2×CO_2_CH_2_), 1.15 (6H, t, *J*=7.1 Hz, 2×CH_3_), 1.13 (6H, t, *J*=7.1 Hz, 2×CH_3_). *δ*_C_ (126 MHz: CDCl_3_): 187.42 (CH), 171.45 (C), 170.66 (C), 157.32 (C), 150.12 (C), 146.39 (C), 143.80 (C), 139.39 (C), 132.70 (CH), 122.02 (CH), 121.57 (CH), 118.99 (CH), 117.92 (CH_2_), 117.75 (C), 113.20 (CH), 110.55 (CH), 102.25 (CH), 69.81 (CH_2_), 67.54 (CH_2_), 66.83 (CH_2_), 61.16 (CH_2_), 60.69 (CH_2_), 53.83 (CH_2_), 53.42 (CH_2_), 14.00 (CH_3_), 13.94 (CH_3_). *m*/*z* (ESI): Found: 695.2773. C_34_H_44_O_12_N_2_ requires (M+Na)^+^, 695.2786. υ_max_ (ATR)/cm^−1^: 2987 (CH), 2868 (CH), 1745 (CO_2_), 1732 (CHO), 1666 (C=C).

#### 4.2.11 Synthesis of nitroaryl **13**

Fuming nitric acid (0.10 ml of a stock solution in dichloromethane (0.11 ml in 1.0 ml)) was added slowly to a stirred solution of aldehyde **12** (165 mg, 0.24 mmol, 1.0 equiv) and acetic acid (0.2 ml) in dry dichloromethane (2.0 ml) at 0 °C under argon. The resulting deep red coloured solution was stirred at 0 °C for 45 min then pour into aqueous potassium carbonate. The product was extracted with dichloromethane (2×10 ml) and the combined organics washed with brine (2×50 ml). The organic layer was dried over magnesium sulfate and concentrated under vacuum. The resulting residue was purified by column chromatography on silica elution EtOAc:Hexane 1:1 to give aldehyde **13** as a bright yellow solid (100 mg, 57%). mp 125–126 °C. *δ*_H_ (500 MHz: CDCl_3_): 10.27 (1H, s, CHO), 7.82 (1H, dd, *J*=9.0, 2.5 Hz, H-4), 7.69 (1H, d, *J*=2.5 Hz, H-6), 7.28 (1H, s, H-6′), 6.68 (1H, d, *J*=9.0 Hz, H-3), 6.28 (1H, s, H-3′), 6.02 (1H, ddt, *J*=17.2, 10.5, 5.2 Hz, C*H*=CH_2_), 5.39 (1H, ddd, *J*=17.3, 3.0, 1.5 Hz, CH=C*H*_*A*_H_B_), 5.30 (1H, ddd, *J*=10.6, 2.6, 1.3 Hz, CH=CH_A_*H*_*B*_), 4.55 (2H, dt, *J*=5.2, 1.4 Hz, OCH_2_), 4.28–4.26 (2H, m, OC*H*_2_CH_2_O), 4.25–4.23 (2H, m, OCH_2_C*H*_2_O), 4.20 (4H, s, 2×NCH_2_), 4.19 (4H, s, 2×NCH_2_), 4.12–4.07 (8H, m, 4×CO_2_CH_2_), 1.17 (12H, t, *J*=7.1 Hz, 4×CH_3_). *δ*_C_ (126 MHz: CDCl_3_): 187.32 (CH), 170.50 (C), 170.30 (C), 157.54 (C), 148.45 (C), 146.50 (C), 145.50 (C), 143.60 (C), 140.79 (C), 132.67 (CH), 118.42 (CH), 117.99 (CH_2_), 117.90 (C), 115.94 (CH), 111.20 (CH), 108.10 (CH), 102.53 (CH), 69.85 (CH_2_), 67.59 (CH_2_), 67.27 (CH_2_), 61.30 (CH_2_), 61.17 (CH_2_), 53.91 (CH_2_), 53.83 (CH_2_), 14.04 (CH_3_), 14.00 (CH_3_). *m*/*z* (FAB): 717.4 ((M+H)^+^, 70%), 644.2 (100), 599.2 (40), 392.2 (70). Found: 717.2748. C_34_H_43_O_14_N_3_ requires (M+H)^+^, 717.2745. υ_max_ (ATR)/cm^−1^: 2982 (CH), 2939 (CH), 1737 (CO_2_), 1730 (CHO), 1662 (C=C), 1518 (CNO_2_), 1327 (CNO_2_).

#### 4.2.12 Synthesis of benzofuran **14**

Osmium tetroxide (47 μl, 0.007 mmol, 0.03 equiv, 4% solution in water) was added to a solution of allyl ether **13** (179 mg, 0.249 mmol), sodium periodate (213 mg, 0.996 mmol, 4.0 equiv) and 2,6-lutidine (56 μl, 0.498 mmol, 2.0 equiv) in dioxane:water (3:1) (4 ml). The solution was stirred under argon at rt overnight. The resulting bright yellow solution was extracted into EtOAc (2×20 ml). The combined organic layers were washed with brine (2×50 ml) and 1 M HCl (50 ml) dried over magnesium sulfate and concentrated under vacuum. The residue was dissolved in acetic acid (2 ml) then heated to 90 °C under argon for 1 h. After cooling the solution was neutralised with saturated sodium bicarbonate solution and extracted into EtOAc (2×20 ml). The combined organic layer was washed with brine (2×30 ml), dried over magnesium sulfate and concentrated under vacuum. The residue was recrystallised twice from MeOH to give benzofuran **14** as a bright yellow solid (34 mg, 19% over two steps). mp 154–155 °C. *δ*_H_ (500 MHz: CDCl_3_): 9.71 (1H, s, CHO), 7.83 (1H, dd, *J*=8.9, 2.5 Hz, H-4), 7.73 (1H, d, *J*=2.5 Hz, H-6), 7.42 (1H, d, *J*=0.6 Hz, H-3′ Furan ring), 7.10 (1H, s, H-4′), 6.97 (1H, s, H-7′), 6.70 (1H, d, *J*=9.0, H-3), 4.36–4.30 (4H, m, OCH_2_CH_2_O), 4.22 (4H, s, 2×NCH_2_), 4.20 (4H, s, 2×NCH_2_), 4.09 (4H, q, *J*=7.1 Hz, 4×CO_2_CH_2_), 4.06 (4H, q, *J*=7.1 Hz, 2×CO_2_CH_2_), 1.18 (6H, t, *J*=7.1 Hz, 2×CH_3_), 1.14 (6H, t, *J*=7.1 Hz, 2×CH_3_). *δ*_C_ (126 MHz: CDCl_3_): 178.43 (CH), 170.67 (C), 170.35 (C), 152.89 (C), 152.61 (C), 148.82 (C), 148.55 (C), 145.35 (C), 143.11 (C), 140.93 (C), 120.07 (C), 118.40 (CH), 180.08 (CH), 116.11 (CH), 108.10 (CH), 105.84 (CH), 101.67 (CH), 67.65 (CH_2_), 67.63 (CH_2_), 61.25 (CH_2_), 61.07 (CH_2_), 53.83 (CH_2_), 14.04 (CH_3_), 13.97 (CH_3_). *m*/*z* (EI): 701.1 (M^+^, 40%), 628.1 (100), 600.1 (15). Found: 701.2431. C_33_H_39_O_14_N_3_ requires (M^+^), 701.2432. υ_max_ (ATR)/cm^−1^: 2983 (CH), 2937 (CH), 1739 (CO_2_), 1514 (CNO_2_), 1329 (CNO_2_).

#### 4.2.13 Synthesis of thiohydantoin **16**

Thiocarbonyl diimidazole (5.87 g, 32.96 mmol, 1.1 equiv) was added to a solution of 1-amino-3-azidopropane^37^ **15** (3.00 g, 29.96 mmol, 1.0 equiv) in dry CHCl_3_ (50 ml) at 0 °C. The solution was warmed to rt and stirred at rt for a further three hours. After this time glycine methyl ester hydrochloride (4.51 g, 35.95 mmol, 1.2 equiv) and triethylamine (5.0 ml, 35.95 mmol, 1.2 equiv) were added and the solution heated to 70 °C overnight under an atmosphere of argon. After cooling the solution was washed with 1M HCl (2×100 ml) dried over sodium sulfate and concentrated under vacuum. The deep purple residue was passed through a plug of silica elution EtOAc:Petroleum ether to give *thiohydantoin* **16** as a orange solid (2.84 g, 47%). mp 62–65 °C. *δ*_H_ (500 MHz: CDCl_3_): 7.30 (1H, broad s, NH), 4.10 (2H, s, CH_2_), 3.92 (2H, t, *J*=7.0 Hz, NCH_2_), 3.39 (2H, t, *J*=6.6 Hz, N_3_CH_2_), 2.01–1.93 (2H, m, CH_2_C*H*_*2*_CH_2_). *δ*_C_ (126 MHz: CDCl_3_): 184.79 (C), 171.44 (C), 49.02 (CH_2_), 48.38 (CH_2_), 38.85 (CH_2_), 27.03 (CH_2_). *m*/*z* (CI): 200.0 ((M+H)^+^, 100%), 157.0 (M−N_3_, 100). Found: 200.0603. C_6_H_9_ON_5_S requires (M+H^+^), 200.0606. υ_max_ (ATR)/cm^−1^: 3219 (NH), 2928 (CH), 2090 (N_3_), 1705 (NCO), 1516 (NCS).

#### 4.2.14 Synthesis of NitroAzidoFuraRed tetraethyl ester **17**

Piperidine (1 drop) was added to a solution benzofuran **14** (56 mg, 0.079 mmol) and thiohydantoin **16** (24 mg, 0.119 mmol, 1.5 equiv) in EtOH (3 ml). The deep red coloured solution was heated to 70 °C overnight under an atmosphere of argon. After cooling the resulting solid filtered and washed with EtOH to give the azide **17** a bright red solid (47 mg, 67%). mp 162–165 °C. *δ*_H_ (500 MHz: CDCl_3_): 9.40 (1H, s, NH), 7.85 (1H, dd, *J*=8.9, 2.4 Hz, H-4), 7.73 (1H, d, *J*=2.4 Hz, H-6), 7.03 (1H, d, *J*=0.6 Hz, H-7′), 7.01 (1H, s, H-4′), 6.92 (1H, s, H-3′ Furan ring), 6.69 (1H, d, *J*=8.9, H-3), 6.56 (1H, s, CH=C), 4.37–4.30 (4H, m, OCH_2_CH_2_O), 4.23 (4H, s, 2×NCH_2_), 4.22 (4H, s, 2×NCH_2_), 4.12 (4H, q, *J*=7.2 Hz, 4×CO_2_CH_2_), 4.07 (4H, q, *J*=7.1 Hz, 2×CO_2_CH_2_), 4.05–4.01 (2H, m, NCH_2_), 3.41 (2H, t, *J*=6.6 Hz, CH_2_N_3_), 2.06–1.99 (2H, m, CH_2_C*H*_*2*_CH_2_), 1.21 (6H, t, *J*=7.1 Hz, 2×CH_3_), 1.15 (6H, t, *J*=7.1 Hz, 2×CH_3_). *δ*_C_ (126 MHz: CDCl_3_): 176.09 (C), 170.79 (C), 170.39 (C), 163.23 (C), 152.18 (C), 150.95 (C), 148.59 (C), 148.54 (C), 145.34 (C), 141.05 (C), 140.90 (C), 124.44 (C), 121.75 (C), 118.39 (CH), 116.05 (CH), 112.88 (CH), 108.10 (CH), 105.15 (CH), 101.59 (CH), 100.23 (CH), 67.66 (CH_2_), 61.27 (CH_2_), 61.09 (CH_2_), 53.83 (CH_2_), 48.97 (CH_2_), 38.84 (CH_2_), 27.26 (CH_2_), 14.05 (CH_3_), 13.99 (CH_3_). *m*/*z* (ESI): 905.2702 (M+Na)^+^, 756.2500, 724.2245. Found: 905.2623. C_39_H_46_O_14_N_8_S requires ((M+Na)^+^), 905.2746. υ_max_ (ATR)/cm^−1^: 3315 (NH), 2978 (CH), 2939 (CH), 2098 (N_3_), 1745 (CO_2_), 1716 (C=O), 1510 (CNO_2_).

#### 4.2.15 Synthesis of NitroAzidoFuraRed

Potassium hydroxide (0.3 ml of a 0.5M solution) was added to a suspension of tetraethyl ester **17** (7.6 mg, 8.6 μmol) in dioxane (1 ml). The solution was stirred at rt under an atmosphere of argon and shielded from the light for 3 h then water (1 ml) was added and the solution stirred for a further 1 h. After this time the red coloured solution was further diluted with water (15 ml), acidified with 1M hydrochloric acid and extracted into EtOAc (2×10 ml). The combined organic layers were then washed with brine (2×30 ml), dried over sodium sulfate and concentrated under vacuum to give the *tetraacid* **NitroAzidoFuraRed** as a red coloured solid (5.7 mg, 86%). *δ*_H_ (400 MHz: d-3 MeCN): 10.11 (1H, s, NH), 7.85 (1H, dd, *J*=9.0, 2.5 Hz, H-4), 7.78 (1H, d, *J*=2.5 Hz, H-6), 7.28 (1H, s, H-7′), 7.17 (1H, s, H-4′), 7.11 (1H, s, H-3′ Furan ring), 6.81 (1H, d, *J*=9.0, H-3), 6.59 (1H, s, CH=C), 4.43–4.39 (2H, m, OC*H*_2_CH_2_O), 4.37–4.32 (2H, m, OCH_2_C*H*_2_O), 4.25 (4H, s, 2×NCH_2_), 4.17 (4H, s, 2×NCH_2_), 3.97 (2H, t, *J*=6.8 Hz, NCH_2_), 3.44 (2H, t, *J*=6.6 Hz, CH_2_N_3_), 1.96 (presumed 2H, m, obscured by MeCN). HRMS obtained on **NitroAzidoFuraRed-FFKDEL** derivative below.

#### 4.2.16 Synthesis of azide **18**

Benzaldehyde (0.13 ml, 1.3 mmol, 1.3 equiv) and piperidine (2 drops) were added to a solution of thiohydantoin **16** (200 mg, 1.00 mmol) in EtOH (3 ml). The solution was heated to 70 °C for 4 h under an atmosphere of argon. After cooling to rt the solvent was removed under vacuum and the residue purified by column chromatography on a 10 g SNAP ultra cartridge eluting 100% hexane increasing to 30% EtOAc/70% Hexane to give the azide as a bright yellow solid (252 mg, 88%). mp 92–96 °C. *δ*_H_ (500 MHz: CDCl_3_): 8.93 (1H, broad s, NH), 7.49–4,39 (5H, m, Ar–H), 6.76 (1H, s, CH), 4.01 (2H, t, *J*=7.0 Hz, NCH_2_), 3.40 (2H, t, *J*=6.7 Hz, N_3_CH_2_), 2.01 (2H, quin, *J*=6.8 Hz, CH_2_). *δ*_C_ (126 MHz: CDCl_3_): 177.92 (C), 163.63 (C), 132.66 (C), 129.86 (CH), 129.51 (CH), 129.10 (CH), 126.20 (C), 113.76 (CH), 48.92 (CH_2_), 38.91 (CH_2_), 27.19 (CH_2_). *m*/*z* (ESI): 310.0721 (M+Na)^+^. Found: 310.0721. C_13_H_13_ON_5_SNa requires ((M+Na)^+^), 310.0733. υ_max_ (ATR)/cm^−1^: 3342 (NH), 2947 (CH), 2096 (N_3_), 1712 (NCO), 1464 (NCS).

#### 4.2.17 Synthesis of azide **19**

Potassium carbonate (326 mg, 2.35 mmol, 1.5 equiv) was added to a solution of bromomethyl acetate (0.23 ml, 2.35 mmol, 1.5 equiv) and azide **18** (450 mg, 1.57 mmol) in DMF (6 ml). The solution was stirred at rt overnight, diluted with water (30 ml) and extracted twice into EtOAc (15 ml). The combined organic layer was washed with brine (3×30 ml), dried over magnesium sulfate and concentrated under vacuum. The residue was purified by column chromatography on silica eluting EtOAc:Hexane 1:2 to give the azide as a pale brown solid (240 mg, 42%). mp 70–74 °C. *δ*_H_ (500 MHz: CDCl_3_): 8.14–8.12 (2H, m, Ar–H), 7.42–4,34 (3H, m, Ar–H), 7.00 (1H, s, CH), 5.91 (2H, s, OCH_2_), 3.63 (2H, t, *J*=6.9 Hz, NCH_2_), 3.34 (2H, t, *J*=6.5 Hz, N_3_CH_2_), 2.12 (3H, s, CH_3_), 1.87 (2H, quin, *J*=6.8 Hz, CH_2_). *δ*_C_ (126 MHz: CDCl_3_): 170.45 (C), 169.57 (C), 161.60 (C), 137.48 (C), 133.91 (C), 131.95 (CH), 130.03 (CH), 128.58 (CH), 125.40 (CH), 61.55 (CH_2_), 48.37 (CH_2_), 38.12 (CH_2_), 28.03 (CH_2_), 20.59 (CH_3_). *m*/*z* (EI): 359.3 (M^+^, 8%), 276.2 (30), 204.1 (100). Found: 359.1055. C_16_H_17_O_3_N_5_S requires (M^+^), 359.1052. υ_max_ (ATR)/cm^−1^: 3026 (CH), 2943 (CH), 2098 (N_3_), 1747 (CO_2_), 1710 (NCO), 1494 (NCS).

#### 4.2.18 Synthesis of alkyne **20**

DMAP, (56 mg, 0.46 mmol, 0.1 equiv) was added to a solution of EDCI (1.15 g, 6.00 mmol, 1.3 equiv), 4-pentynoic acid (500 mg, 5.10 mmol, 1.1 equiv) and l-phenylalanine methyl ester hydrochloride (1.00 g, 4.62 mmol) in dry CH_2_Cl_2_ (20 ml). After stirring at rt overnight under argon the solution was diluted with CH_2_Cl_2_ (10 ml), washed with 1M HCl (30 ml) then saturated NaHCO_3_ solution (30 ml). The solution was dried ove magnesium sulfate and concentrated under vacuum to give the alkyne as a white solid (698 mg, 58%). mp 88–90 °C. *δ*_H_ (500 MHz: CDCl_3_): 7.32–7.24 (3H, m, Ar–H), 7.13–7.11 (2H, m, Ar–H), 6.15 (1H, d, *J*=7.7 Hz, NH), 4.92 (1H, dt, *J*=7.8, 5.8 Hz, CH), 3.74 (3H, s, OMe), 3.18 (1H, dd, *J*=13.9, 5.8 Hz, C*H*_*A*_H_B_), 3.11 (1H, dd, *J*=13.9, 5.8 Hz, CH_A_*H*_*B*_), 2.53–2.49 (2H, m, CH_2_), 2.44–2.39 (2H, m, CH_2_), 1.98 (1H, t, *J*=2.6 Hz, CH). *δ*_C_ (126 MHz: CDCl_3_): 171.90 (C), 170.37 (C), 135.74 (C), 129.23 (CH), 128.51 (CH), 127.08 (CH), 82.72 (C), 69.33 (CH), 53.10 (CH), 52.27 (CH_3_), 37.83 (CH_2_), 35.08 (CH_2_), 14.61 (CH_2_). *m*/*z* (EI): 259.2 (M^+^, 5%), 200 (15), 162.1 (100). Found: 259.1206. C_15_H_17_O_3_N requires (M^+^), 259.1208. υ_max_ (ATR)/cm^−1^: 3309 (Alkyne), 3252 (NH), 1720 (CO_2_), 1643 (CON).

#### 4.2.19 Synthesis of triazole **21**

Tetrakis(acetonitrile)copper(I) hexafluorophosphate (12 mg, 0.033 mmol, 0.2 equiv) was added to a solution of azide **19** (60 mg, 0.167 mmol, 1.0 equiv) and alkyne **20** (130 mg, 0.50 mmol, 3.0 equiv) in degassed DMSO (1 ml). The solution was heated to 60 °C for 1 h. After cooling the solution was quenched with water (25 ml) and extracted twice with CH_2_Cl_2_ (8 ml). The combined organic layers were washed twice with brine (20 ml), dried over sodium sulfate and concentrated under vacuum. The residue was purified by column chromatography on a 10 g SNAP ultra cartridge eluting 100% CH_2_Cl_2_ increasing to CH_2_Cl_2_:6% MeOH to give the triazole as a pale yellow viscous oil (83 mg, 80%). *δ*_H_ (500 MHz: CDCl_3_): 8.15–8.13 (2H, m, Ar–H), 7.50 (1H, s, CH), 7.45–7.38 (3H, m, Ar–H), 7.28–7.20 (3H, m, Ar–H), 7.07–7.05 (2H, m, Ar–H), 7.01 (1H, s, PhC*H*), 6.40 (1H, d, *J*=7.8 Hz, NH), 5.90 (2H, s, OCH_2_), 4.86 (1H, dt, *J*=7.8, 6.1 Hz, NHC*H*), 4.33 (2H, t, *J*=6.9, NCH_2_), 3.69 (3H, s, OMe), 3.60 (2H, t, *J*=6.8, NCH_2_), 3.12 (1H, dd, *J*=13.9, 5.7 Hz, C*H*_*A*_H_B_), 3.06–3.01 (3H, m, CH_A_*H*_*B*_+COCH_2_C*H*_*2*_), 2.61 (2H, t, *J*=7.3, COC*H*_*2*_CH_2_), 2.25 (2H, quin, *J*=6.8 Hz, CH_2_*CH*_*2*_CH_2_), 2.13 (3H, s, CH_3_). *δ*_C_ (126 MHz: CDCl_3_): 171.87 (C), 171.48 (C), 170.57 (C), 169.74 (C), 161.30 (C), 146.45 (C), 137.34 (C), 135.83 (C), 133.83 (C), 132.04 (CH), 130.23 (CH), 129.04 (CH), 128.61 (CH), 128.41 (CH), 126.91 (CH), 125.82 (CH), 121.77 (CH), 61.58 (CH_2_), 53.06 (CH), 52.12 (CH_3_), 47.14 (CH_2_), 37.80 (CH_2_), 37.66 (CH_2_), 35.34 (CH_2_), 29.46 (CH_2_), 21.18 (CH_2_), 20.62 (CH_3_). *m*/*z* (ESI): 641.2137 (M+Na)^+^. Found: 641.2137. C_31_H_34_O_6_N_6_S requires ((M+Na)^+^), 641.2153. υ_max_ (ATR)/cm^−1^: 3300 (NH), 3252 (NH), 1743 (CO_2_), 17,414 (CO_2_), 1664 (CON).

#### 4.2.20 Synthesis of NitroFuraRed-FFKDEL penta-AM

Hunig's base (0.1 ml, 0.57 mmol, 112 equiv) and bromomethyl acetate (0.1 ml, 1.02 mmol, 204 equiv) were added to a solution of NitroAzidoFuraRed (3.9 mg, 0.005 mmol) in dry MeCN (1 ml). The solution was stirred at rt overnight, diluted with CH_2_Cl_2_ (7 ml), washed with 0.5M HCl (10 ml), dried over magnesium sulfate and concentrated under vacuum. The residue was purified by column chromatography on silica eluting EtO_2_:CHCl_3_ (1:1) to give the product as a deep red solid, which was used without further purification. Tetrakis(acetonitrile)copper(I) hexafluorophosphate (∼0.3 mg, 0.0008 mmol, 0.5 equiv) was added to a solution of crude azide obtained from the above AM protection (2 mg, 0.0017 mmol, 1.0 equiv) and alkyne ((4-Pentynoyl-NH)-FFKDEL-COOH) (Eurogentec, Belgium) (4.7 mg, 0.0053 mmol, 3.0 equiv) in degassed DMSO (0.5 ml). The solution was heated to 70 °C under argon for 1 h, cooled to rt and diluted with water (5 ml) then extracted with CH_2_Cl_2_ (2×5 ml). The combined organic layers were concentrated under vacuum and the residue purified by prep-HPLC carried out on a Spectrasystem P2000 using a 250 mm×21.2 mm Gemini-NX 10 micron C-18 column maintained at 25 °C and eluted 0.1% TFA (90%):acetonitrile (10%) for 10 min followed by a 10–80% gradient at 15 ml/min over a further 40 min. The product containing fractions were concentrated under vacuum to give the *triazole* as a red solid (1.5 mg, 42%). The compound was characterised by LCMS (see ESI).
