# Supplementary material for: Synthesis of an azido-tagged low affinity ratiometric calcium sensor
Source: Tetrahedron. 2015 Dec 23;71(51):9571–8. doi: 10.1016/j.tet.2015.10.052 (PMC4660056; doi:10.1016/j.tet.2015.10.052)
Supplement: Supplementary file 1 [file mmc1.pdf]

Supporting Information for

Synthesis of an Azido-tagged Low Affinity Ratiometric Calcium Sensor

Stuart T. Caldwell,[a] Andrew G. Cairns,[a] Marnie Olson,[b] Susan Chalmers,[b] Mairi Sandison,[b] William Mullen,[c] John G. McCarron[b] and Richard C. Hartley[a*]

[a]*WestCHEM School of Chemistry, University of Glasgow, Glasgow G12 8QQ, U.K.*

[b]*Strathclyde Institute of Pharmacy and Biomedical Sciences, University of Strathclyde, 161 Cathedral Street, Glasgow G4 0RE, U.K.*

[c]*BHF Glasgow Cardiovascular Research Centre, Institute of Cardiovascular and Medical Sciences, College of Medical Veterinary and Life Sciences, University of Glasgow, Glasgow G12 8QQ, UK.*

Contents:

1) LCMS data for NitroFuraRed-FFKDEL S2
2) ^{1}H and ^{13}C NMR spectra for compounds **7** - **14** and **16** - **21** and ^{1}H NMR spectra for compounds **2** - **4**, **6** and **NitroAzidoFuraRed**. S3-34

LCMS data for NitroFuraRed-FFKDEL

LC-MS analysis of the triazole was carried out on a Thermo Acella HPLC system comprising of an autosampler with sampler cooler maintained at 6ºC, a photodiode array detector scanning from 200-600 nm. Samples (5µl) were injected onto a 150 x 3.0mm C_{18} Accucore (Thermo Fisher Scientific) maintained at 40ºC and eluted with a 30-90% gradient of 1.0% formic acid and acetonitrile at 700 µL/min over 0-15 minutes. After passing through the absorbance detector the eluant was directed to the electrospray interface of the Thermo Exactive Orbitrap mass spectrometer. Samples were run in positive ionisation mode, the scan range was from 200-2010 *amu*.

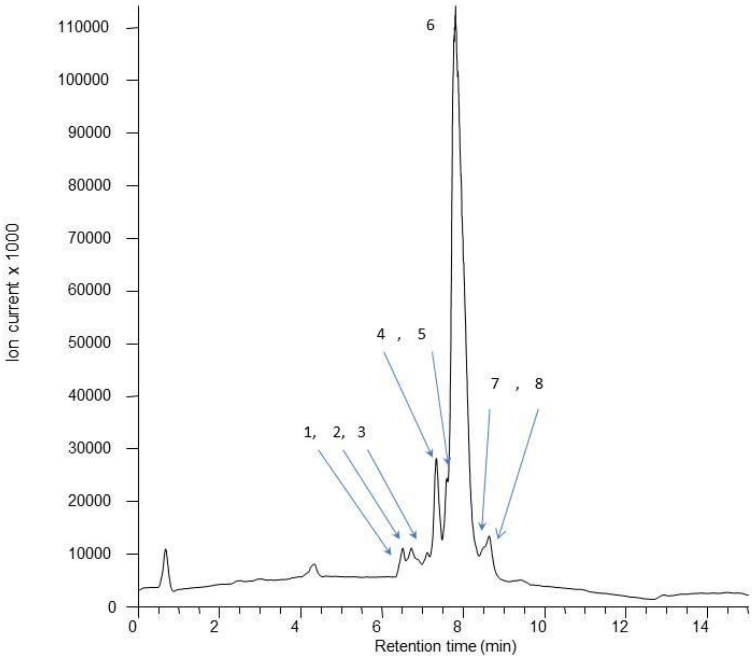

Peak Number	Apex RT	M+1	measured exact mass	Area	%Area
1	6.49	1864.6709	1863.6569	37947995	1.51
2	6.7	1864.6705	1863.6565	28730659	1.15
3	7.1	1920.7140	1919.7000	25021916	1
4	7.33	1920.7095	1919.6955	1.69E+08	6.74
5	7.59	1936.6887	1935.6747	77678888	3.1
**6**	**7.81**	**2008.7030**	**2007.6890***	**2.08E+09**	**82.75**
7	8.49	2008.7106	2007.6966	32980817	1.31
8	8.64	2008.7095	2007.6955	61098367	2.44

*** = Conforms to expected theoretical mass for NitroFuraRed-FFKDEL of 2007.6880 (error 0.5 ppm)**

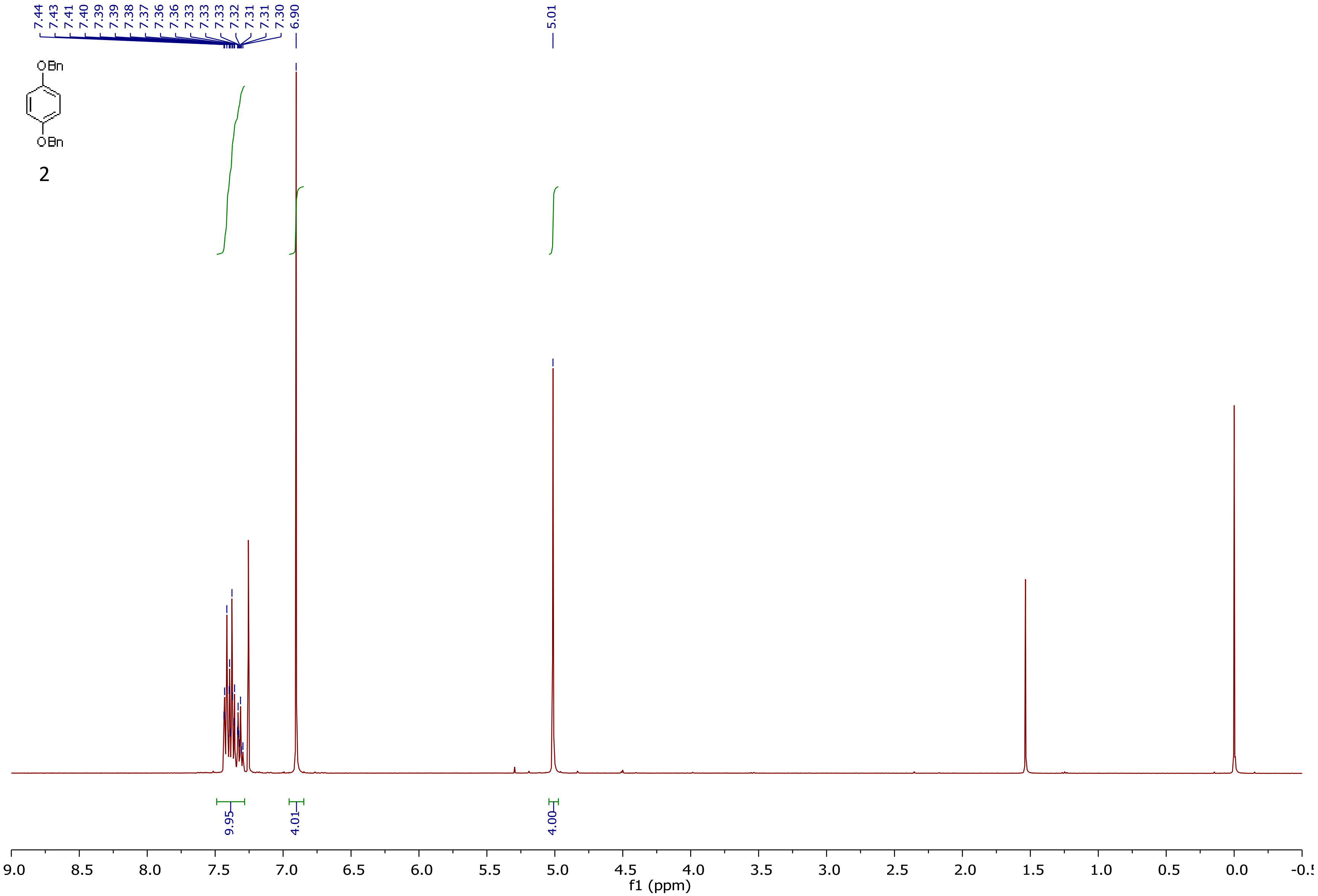
7.44
7.43
7.41
7.40
7.39
7.39
7.38
7.37
7.36
7.36
7.33
7.33
7.33
7.32
7.31
7.31
7.30
6.90
5.01
OBn
OBn
2
9.95
4.01
4.00
9.0
8.5
8.0
7.5
7.0
6.5
6.0
5.5
5.0
4.5
4.0
3.5
3.0
2.5
2.0
1.5
1.0
0.5
0.0
f1 (ppm)

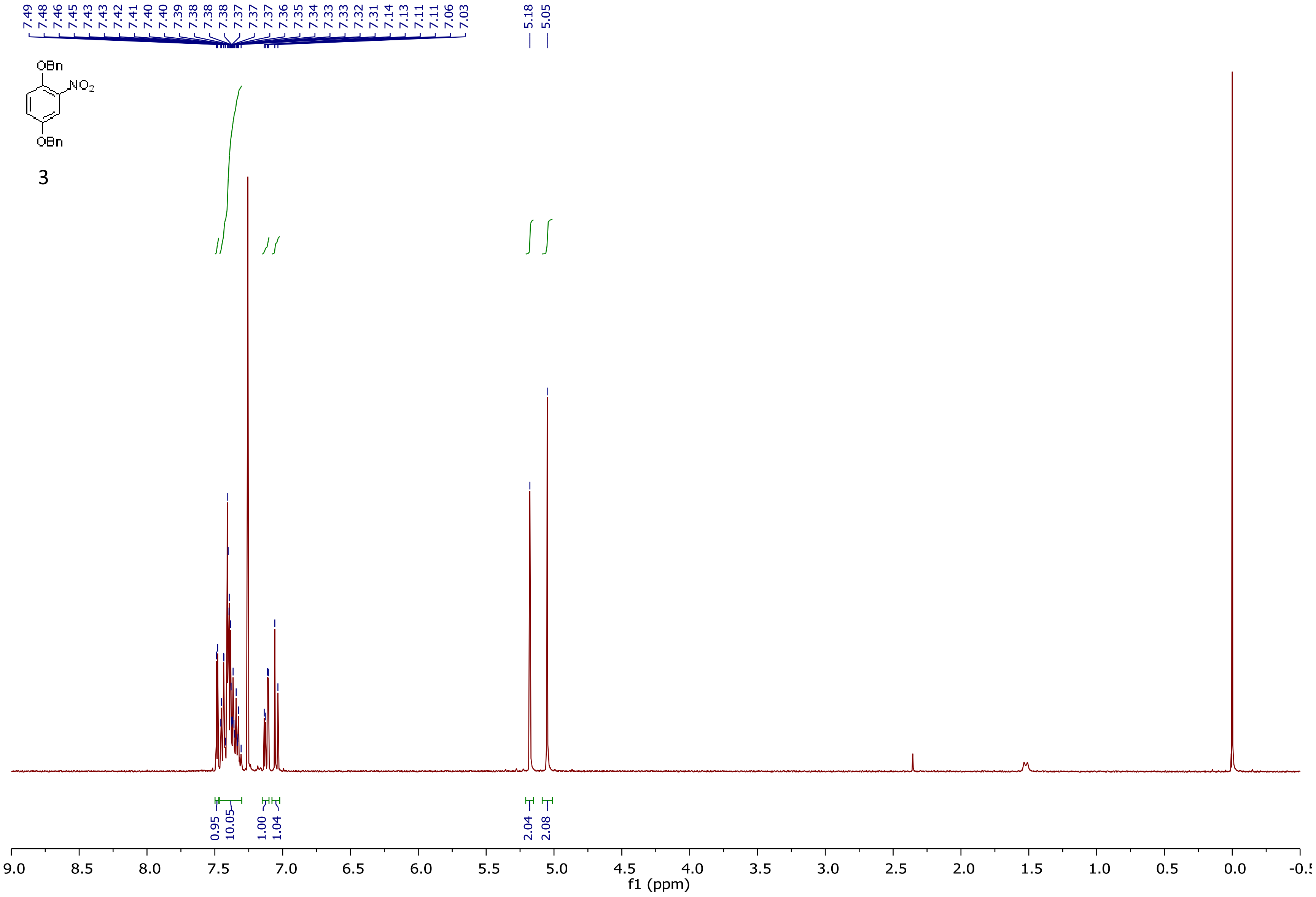

7.49 7.48 7.46 7.45 7.43 7.43 7.42 7.41 7.40 7.40 7.39 7.38 7.38 7.38 7.37 7.37 7.37 7.36 7.35 7.34 7.33 7.33 7.32 7.31 7.14 7.13 7.11 7.11 7.06 7.03
5.18
5.05
OBn
NO2
OBn
3
0.95
10.05
1.00
1.04
2.04
2.08
f1 (ppm)

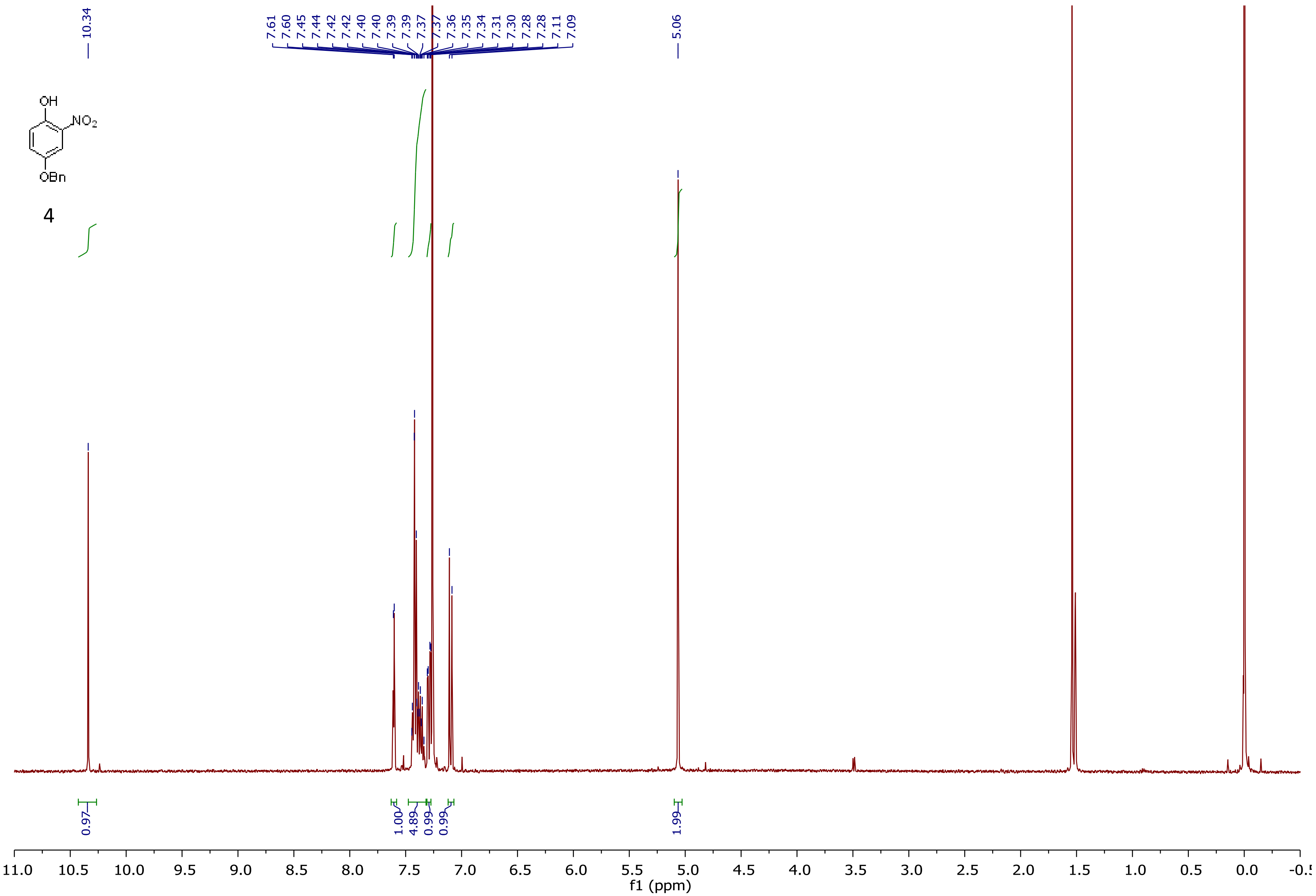

10.34
7.61
7.60
7.45
7.44
7.42
7.42
7.40
7.40
7.39
7.39
7.37
7.37
7.36
7.35
7.34
7.31
7.30
7.28
7.28
7.11
7.09
5.06
OH
NO2
OBn
4
0.97
1.00
4.89
0.99
0.99
1.99
11.0
10.5
10.0
9.5
9.0
8.5
8.0
7.5
7.0
6.5
6.0
5.5
5.0
4.5
4.0
3.5
3.0
2.5
2.0
1.5
1.0
0.5
0.0
f1 (ppm)

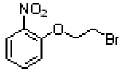
NO2
O
Br

6

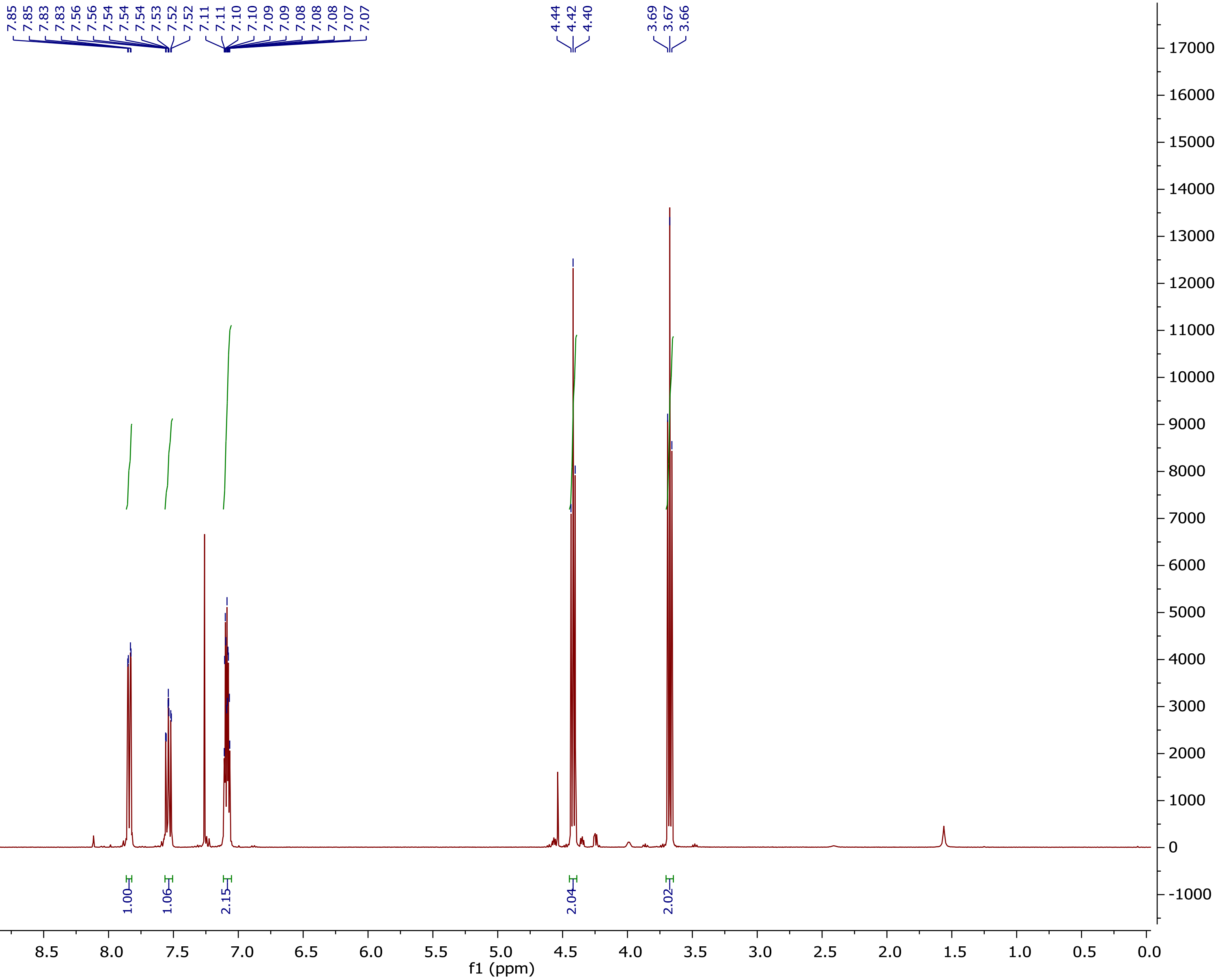
7.85
7.85
7.83
7.83
7.56
7.56
7.54
7.54
7.54
7.53
7.52
7.52
7.11
7.11
7.10
7.10
7.09
7.09
7.08
7.08
7.08
7.07
7.07
4.44
4.42
4.40
3.69
3.67
3.66
1.00
1.06
2.15
2.04
2.02
f1 (ppm)

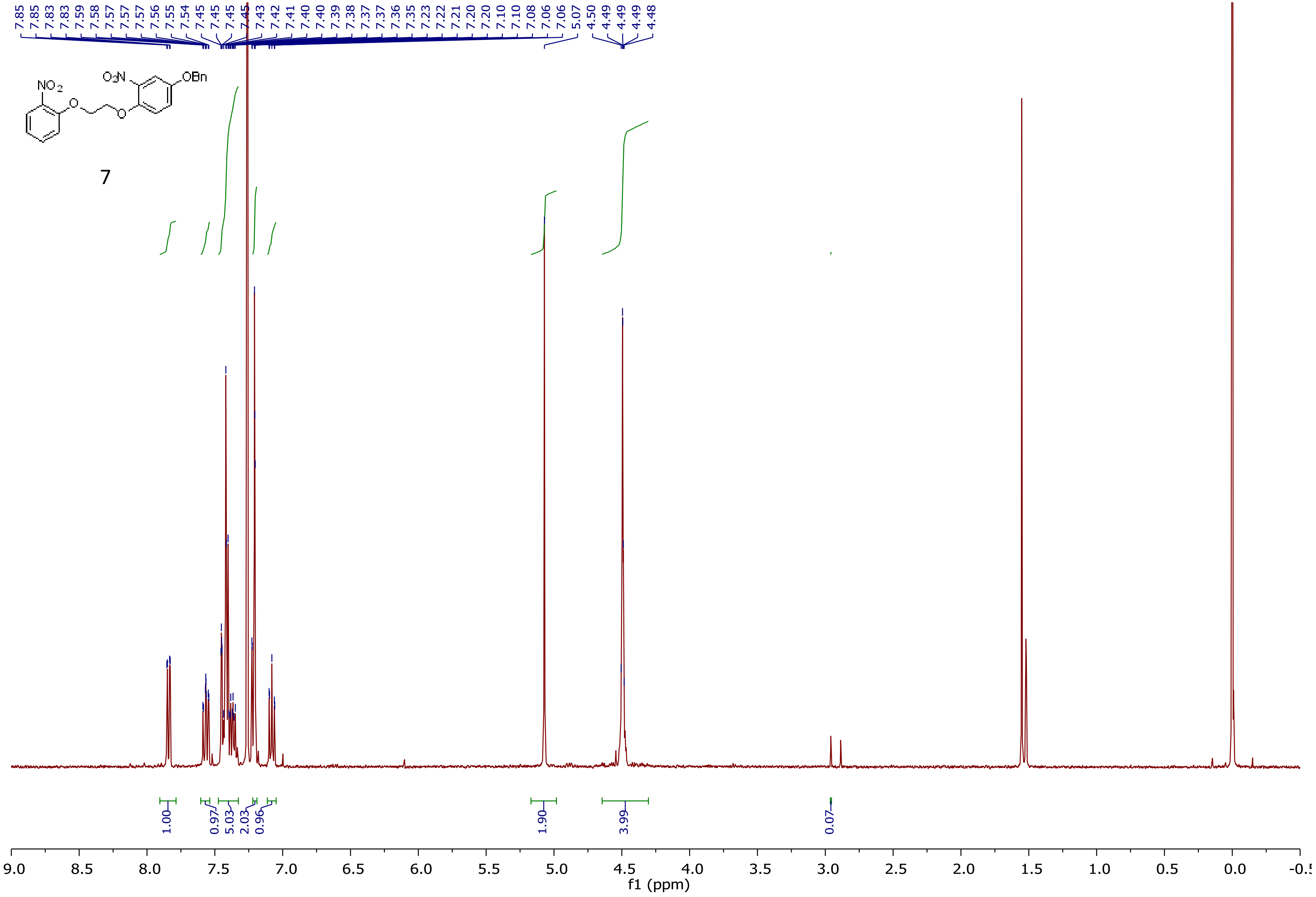

7.85
7.85
7.83
7.83
7.59
7.58
7.57
7.57
7.57
7.56
7.55
7.54
7.45
7.45
7.45
7.45
7.43
7.42
7.41
7.40
7.40
7.39
7.38
7.37
7.37
7.36
7.35
7.23
7.22
7.21
7.20
7.20
7.10
7.10
7.08
7.06
7.06
5.07
4.50
4.49
4.49
4.49
4.48
NO_2
O_2N
OBn
O
O
7
1.00
0.97
5.03
2.03
0.96
1.90
3.99
0.07
9.0
8.5
8.0
7.5
7.0
6.5
6.0
5.5
5.0
4.5
4.0
3.5
3.0
2.5
2.0
1.5
1.0
0.5
0.0
-0.5
f1 (ppm)

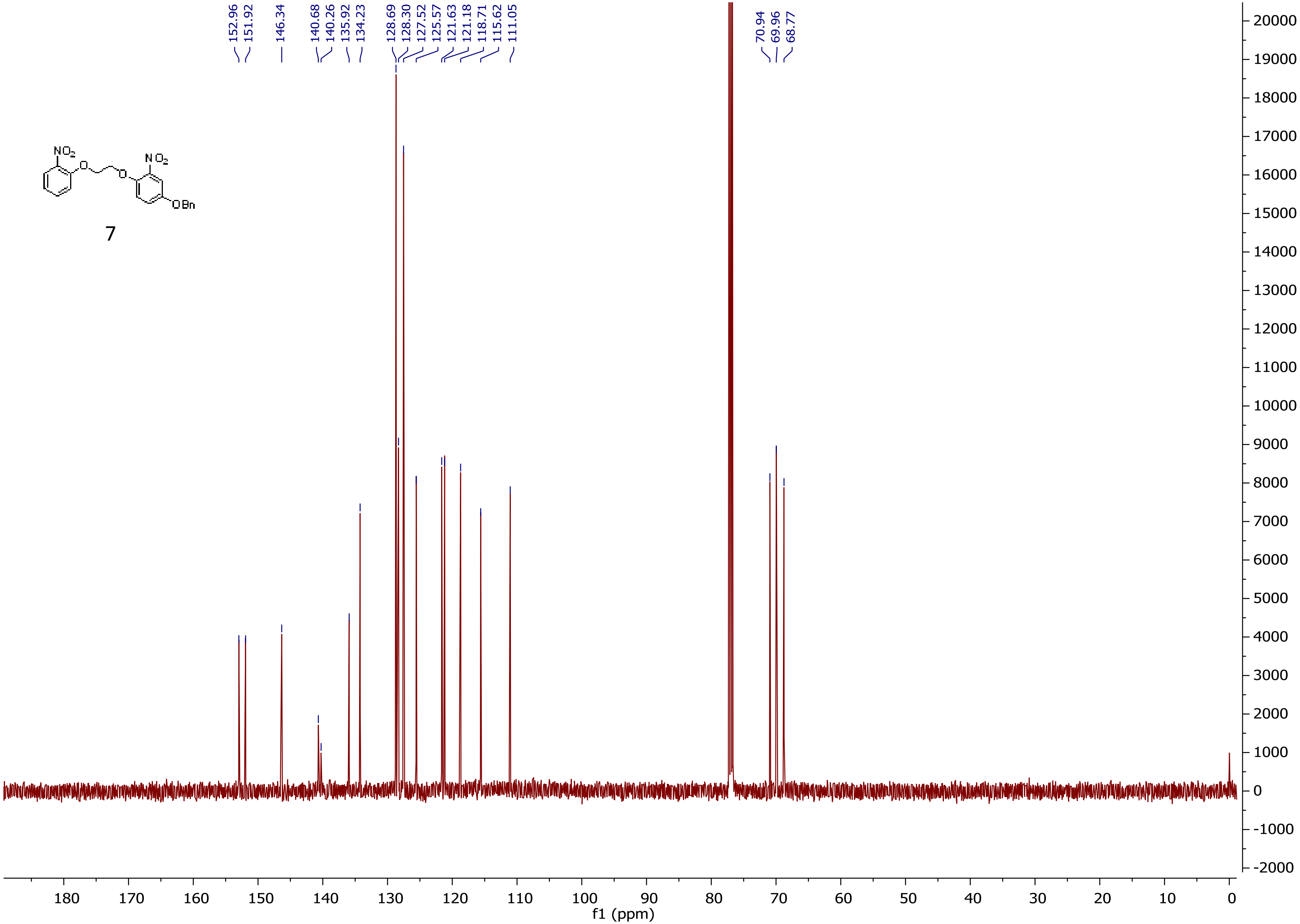
NO2
NO2
O
O
OBn
152.96
151.92
146.34
140.68
140.26
135.92
134.23
128.69
128.30
127.52
125.57
121.63
121.18
118.71
115.62
111.05
70.94
69.96
68.77
f1 (ppm)
180
170
160
150
140
130
120
110
100
90
80
70
60
50
40
30
20
10
0
20000
19000
18000
17000
16000
15000
14000
13000
12000
11000
10000
9000
8000
7000
6000
5000
4000
3000
2000
1000
0
-1000
-2000

7

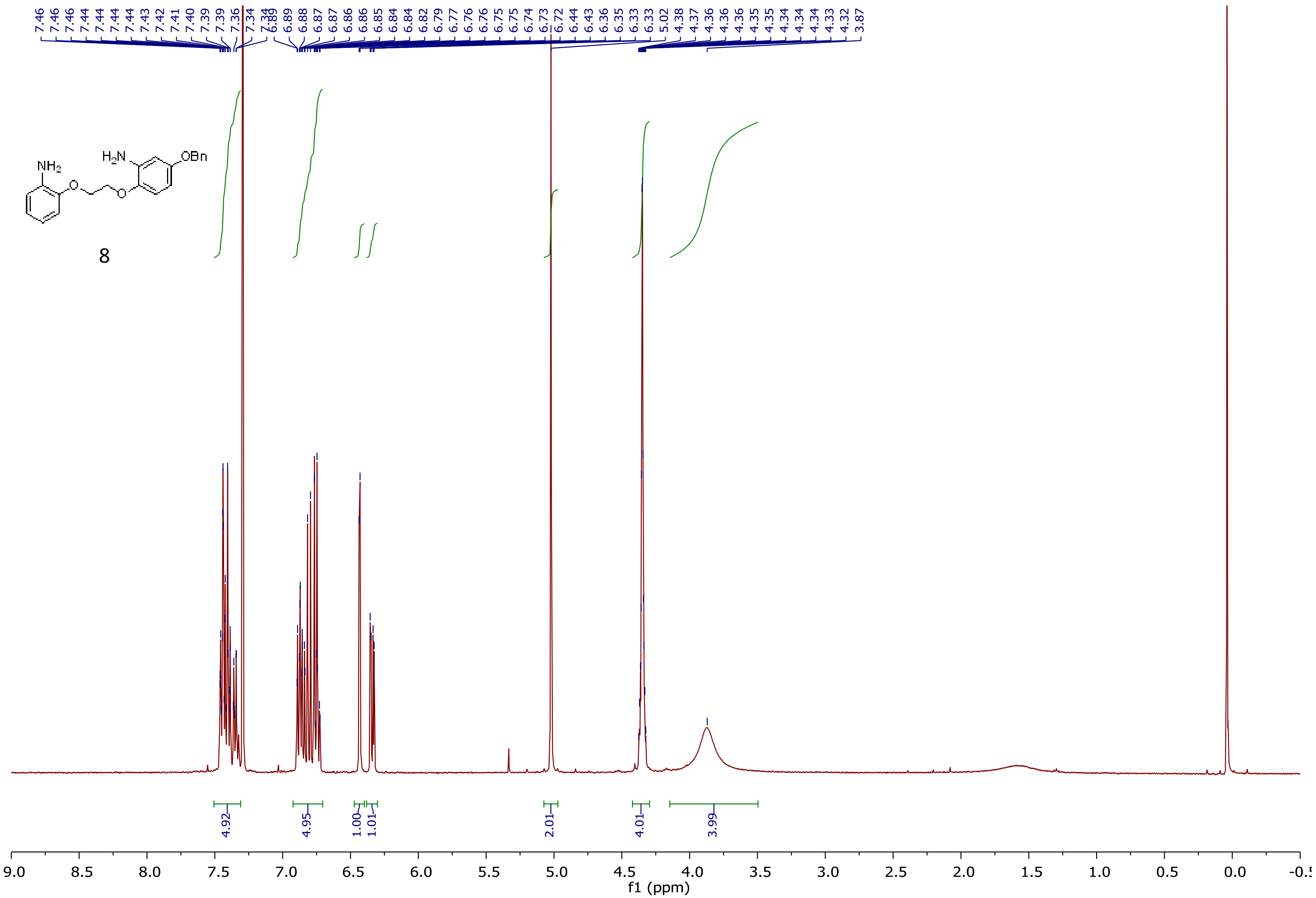

NH2
H2N
OBn
8
f1 (ppm)

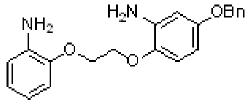

8

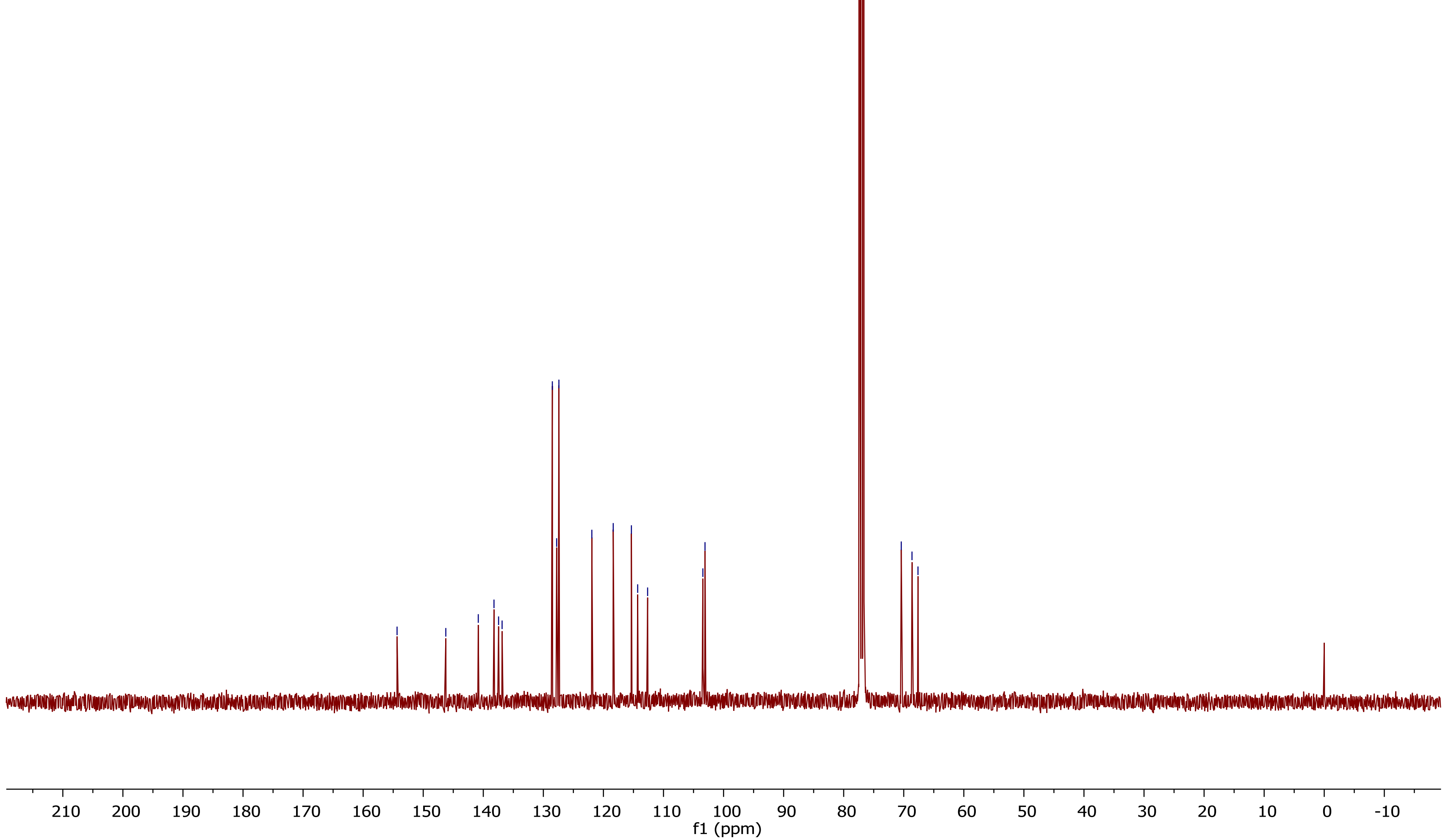

154.35
146.24
140.84
138.22
137.46
136.87
128.51
127.79
127.41
121.93
118.37
115.34
114.30
112.64
103.44
103.09
70.41
68.63
67.63
210
200
190
180
170
160
150
140
130
120
110
100
90
80
70
60
50
40
30
20
10
0
-10
f1 (ppm)

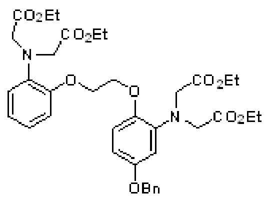
CO2Et
CO2Et
N
O
O
CO2Et
N
CO2Et
OBn

9

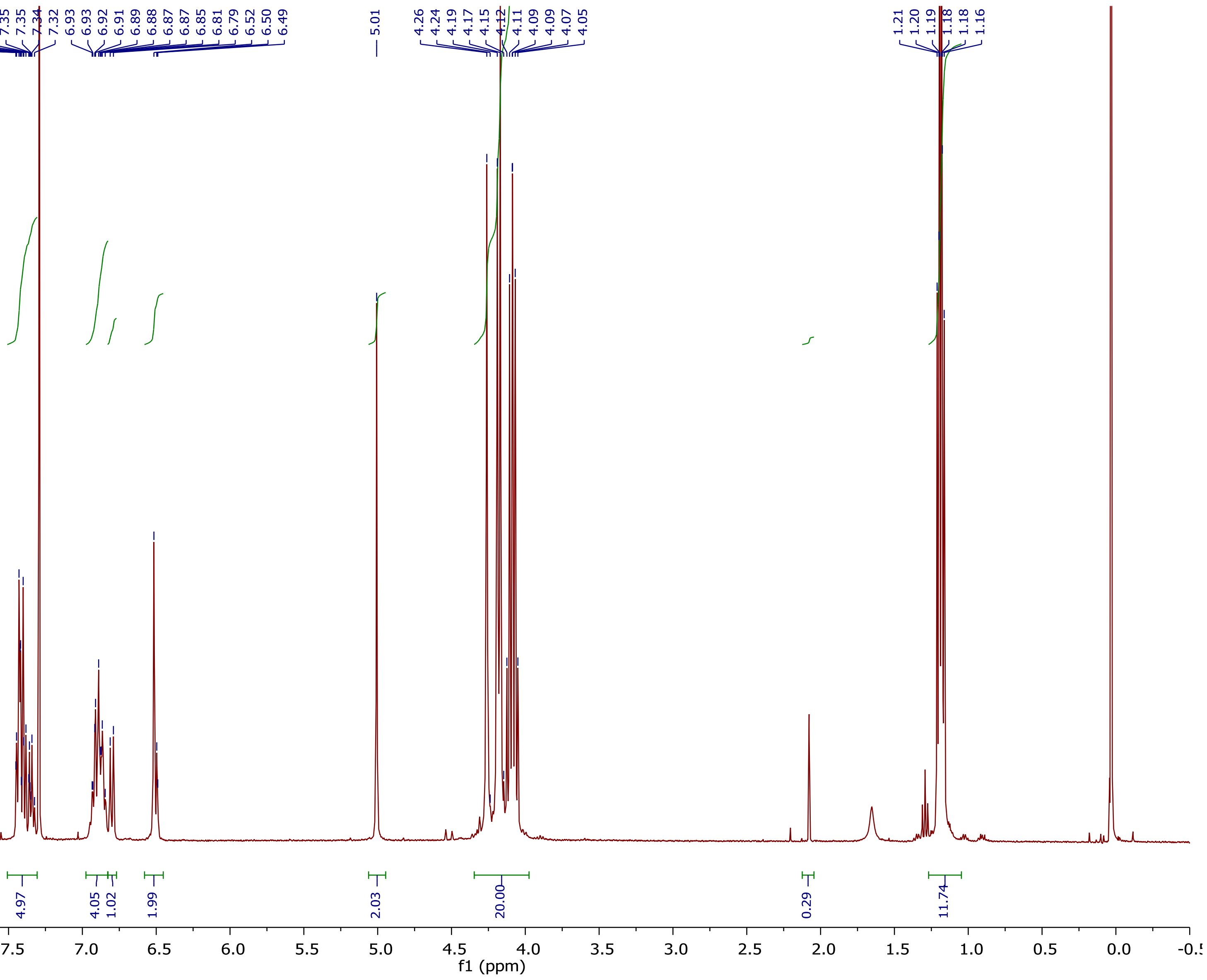
7.45 7.45 7.43 7.42 7.42 7.41 7.40 7.40 7.38 7.36 7.36 7.35 7.35 7.34 7.32 6.93 6.93 6.92 6.91 6.89 6.88 6.87 6.87 6.85 6.81 6.79 6.52 6.50 6.49
5.01
4.26 4.24 4.19 4.17 4.15 4.12 4.11 4.09 4.09 4.07 4.05
1.21 1.20 1.19 1.18 1.18 1.16
4.97
4.05
1.02
1.99
2.03
20.00
0.29
11.74
9.0 8.5 8.0 7.5 7.0 6.5 6.0 5.5 5.0 4.5 4.0 3.5 3.0 2.5 2.0 1.5 1.0 0.5 0.0
f1 (ppm)

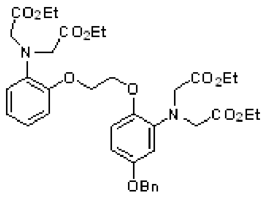
CO2Et
CO2Et
N
O
O
CO2Et
N
CO2Et
OBn

9

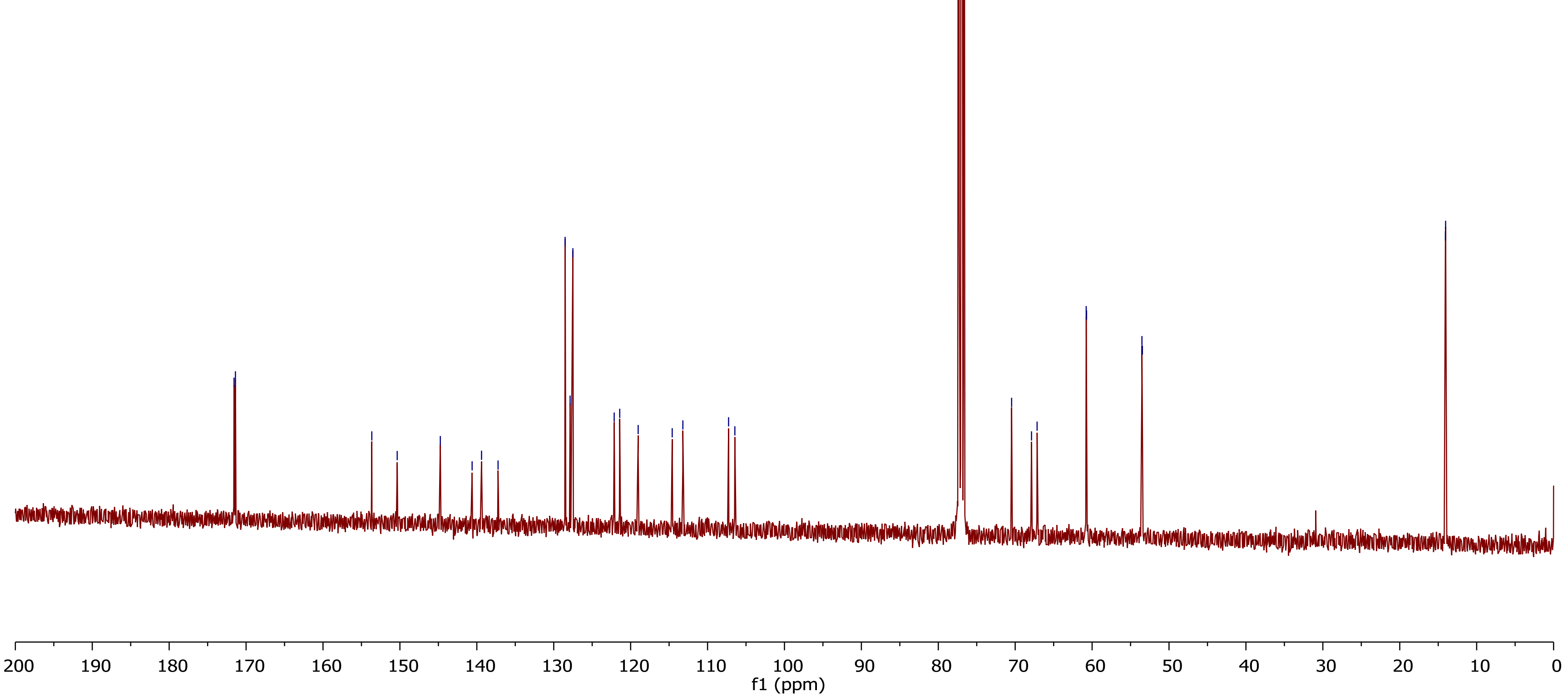
171.57
171.38
153.65
150.35
144.75
140.61
139.39
137.24
128.52
127.87
127.51
122.14
121.42
119.02
114.59
113.21
107.27
106.45
70.46
67.88
67.15
60.77
60.72
53.52
53.46
14.06
14.04
200
190
180
170
160
150
140
130
120
110
100
90
80
70
60
50
40
30
20
10
0
f1 (ppm)

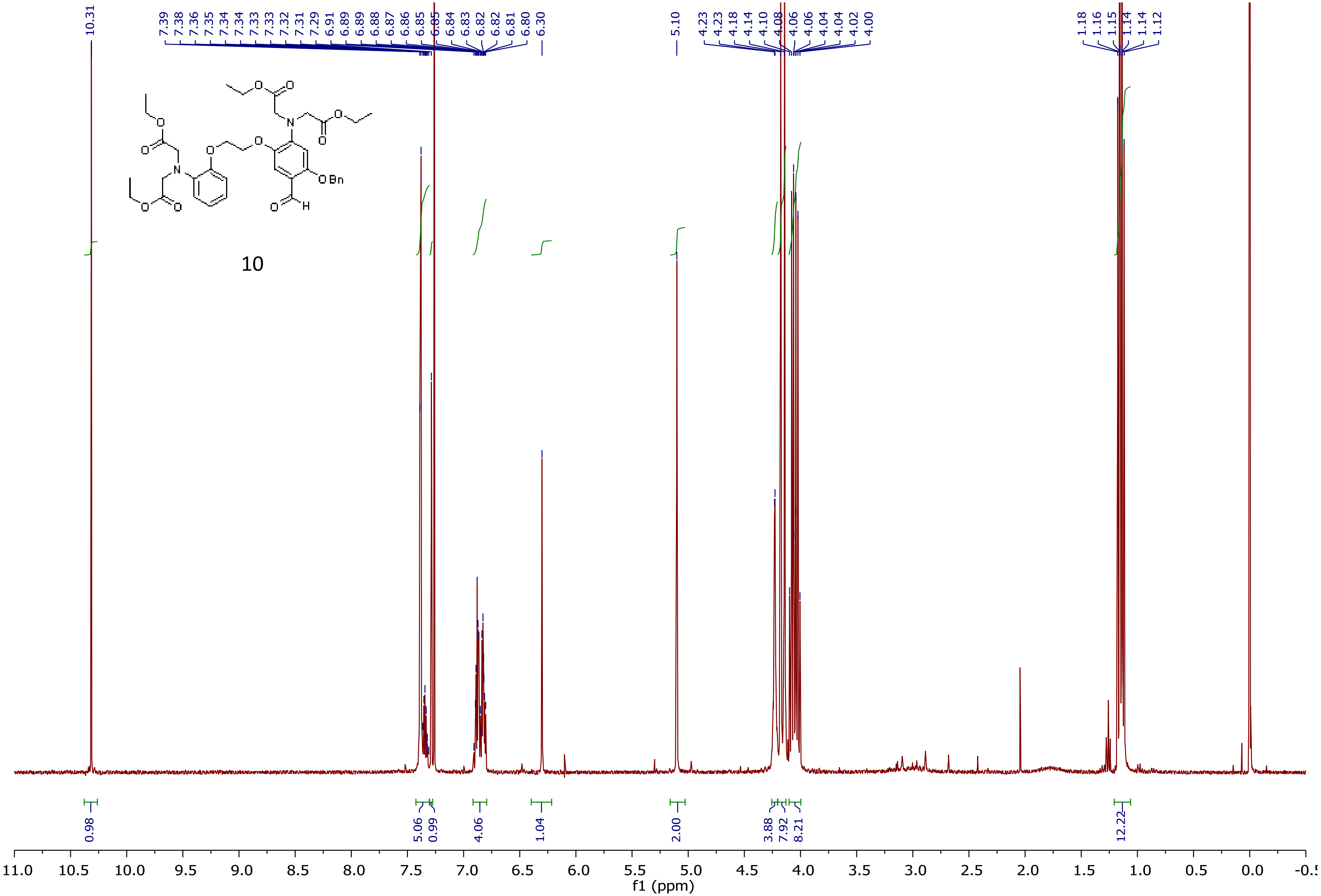
10.31
7.39 7.38 7.36 7.35 7.34 7.34 7.33 7.33 7.32 7.31 7.29 6.91 6.89 6.89 6.88 6.87 6.86 6.85 6.85 6.84 6.83 6.82 6.82 6.81 6.80 6.30
5.10
4.23 4.23 4.18 4.14 4.10 4.08 4.06 4.06 4.04 4.04 4.02 4.00
1.18 1.16 1.15 1.14 1.14 1.12
OBn
H
10
0.98
5.06
0.99
4.06
1.04
2.00
3.88
7.92
8.21
12.22
11.0 10.5 10.0 9.5 9.0 8.5 8.0 7.5 7.0 6.5 6.0 5.5 5.0 4.5 4.0 3.5 3.0 2.5 2.0 1.5 1.0 0.5 0.0
f1 (ppm)

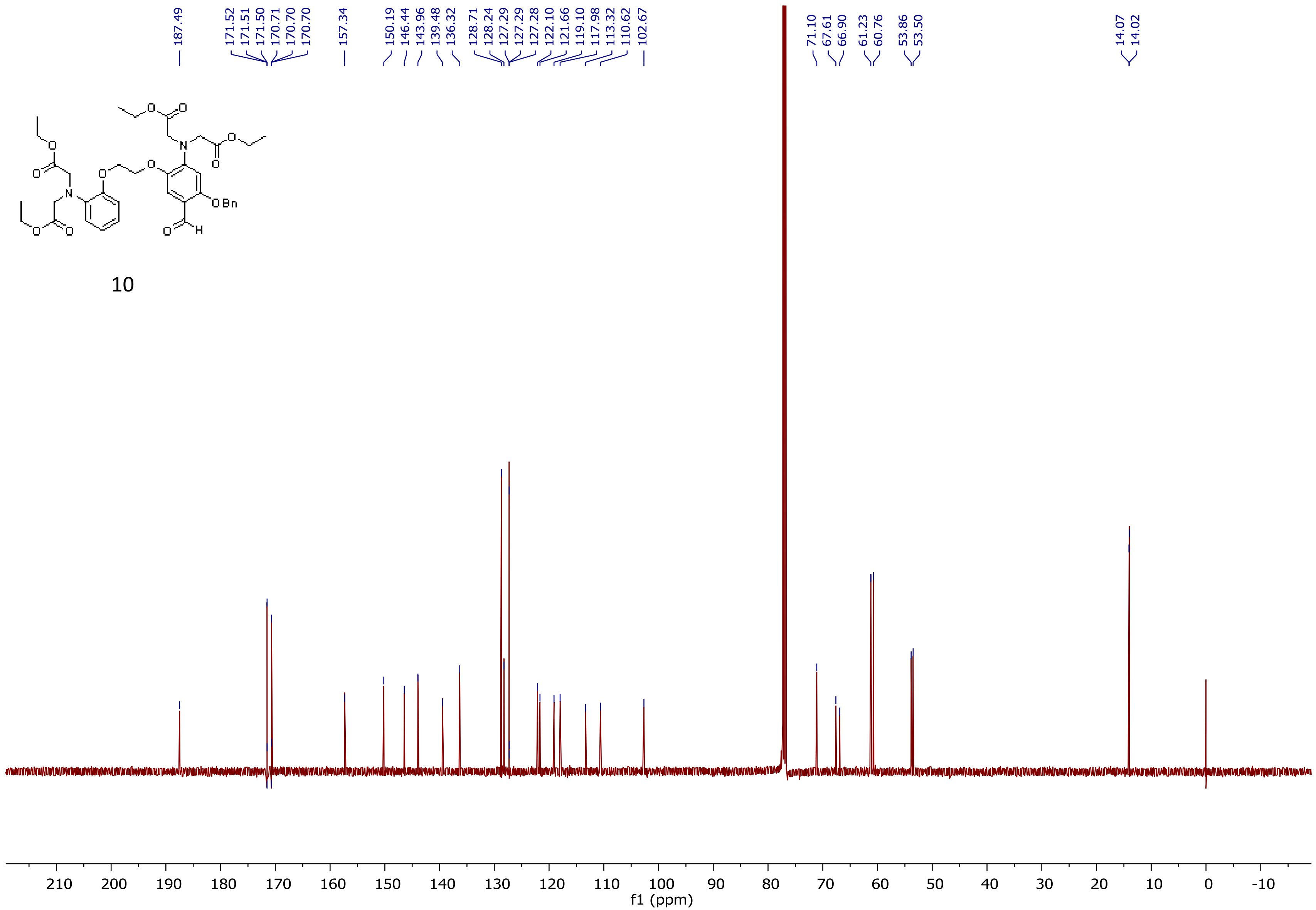

187.49
171.52
171.51
171.50
170.71
170.70
170.70
157.34
150.19
146.44
143.96
139.48
136.32
128.71
128.24
127.29
127.29
127.28
122.10
121.66
119.10
117.98
113.32
110.62
102.67
71.10
67.61
66.90
61.23
60.76
53.86
53.50
14.07
14.02
OBn
10
210 200 190 180 170 160 150 140 130 120 110 100 90 80 70 60 50 40 30 20 10 0 -10
f1 (ppm)

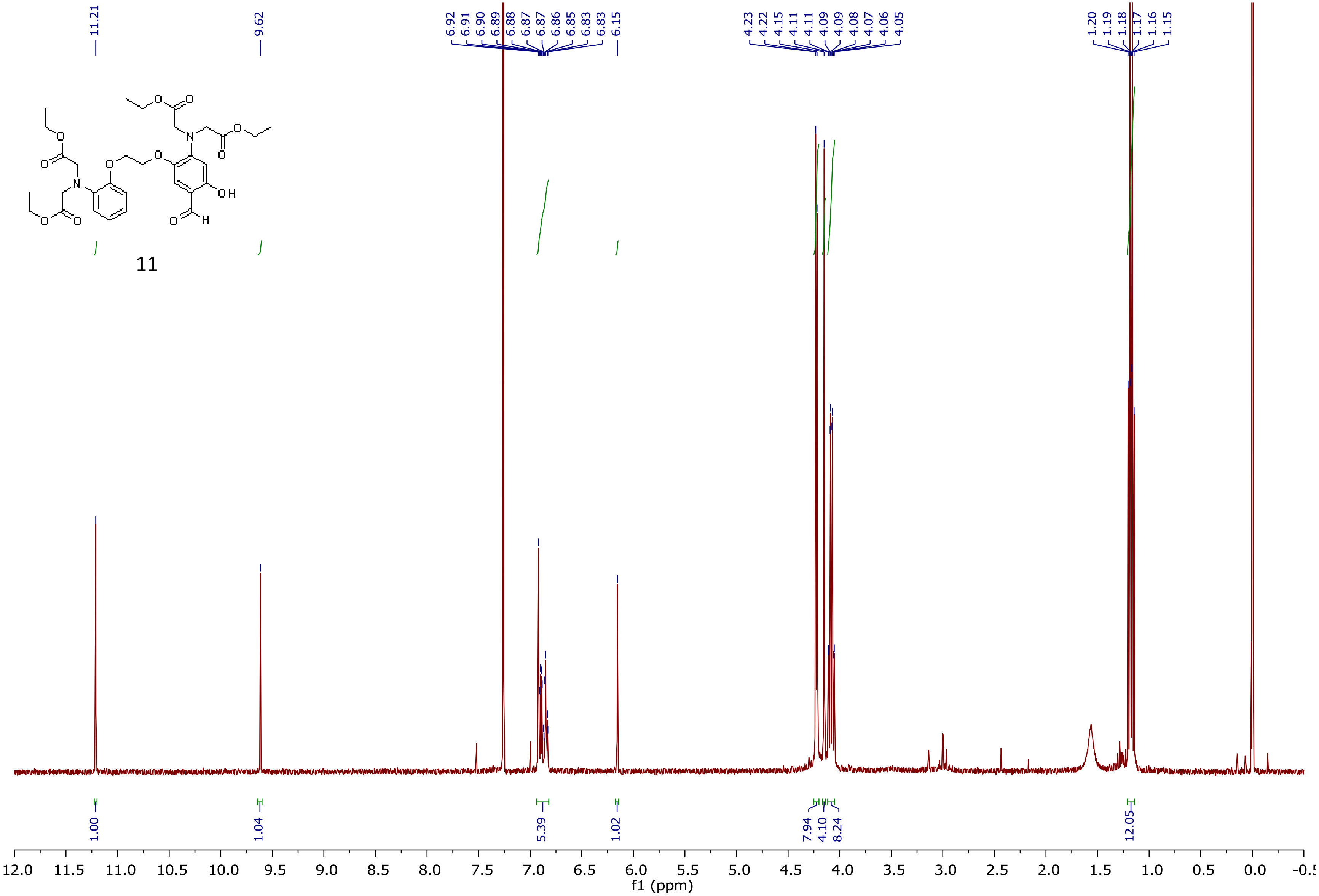

11.21
9.62
6.92 6.91 6.90 6.89 6.88 6.87 6.87 6.86 6.85 6.83 6.83 6.15
4.23 4.22 4.15 4.11 4.11 4.09 4.09 4.08 4.07 4.06 4.05
1.20 1.19 1.18 1.17 1.16 1.15
OH
H
11
1.00
1.04
5.39
1.02
7.94
4.10
8.24
12.05
12.0 11.5 11.0 10.5 10.0 9.5 9.0 8.5 8.0 7.5 7.0 6.5 6.0 5.5 5.0 4.5 4.0 3.5 3.0 2.5 2.0 1.5 1.0 0.5 0.0
f1 (ppm)

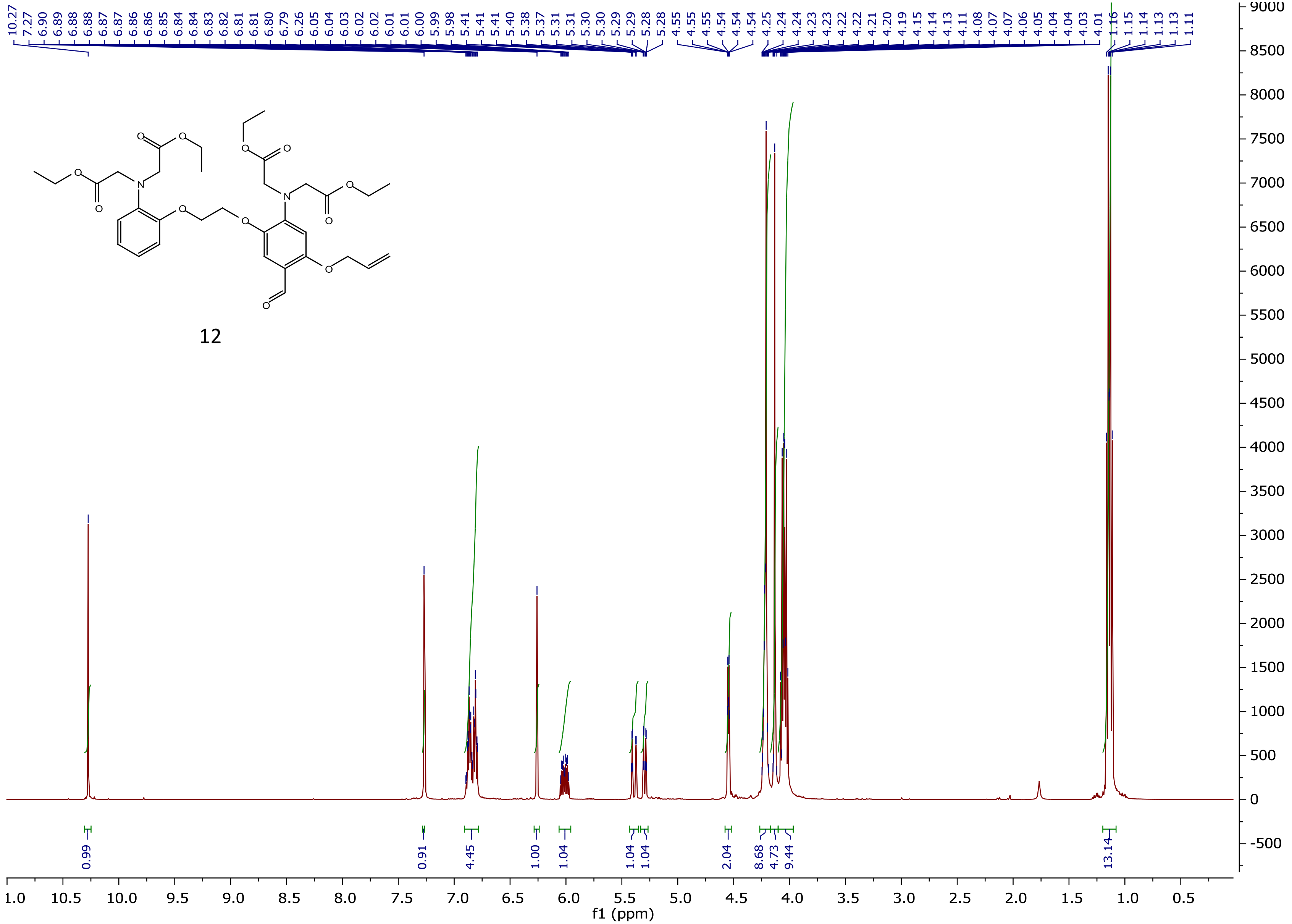

10.27 7.27 6.90 6.89 6.88 6.88 6.87 6.87 6.86 6.86 6.85 6.84 6.84 6.83 6.82 6.81 6.81 6.80 6.79 6.26 6.05 6.04 6.03 6.02 6.02 6.01 6.01 6.00 5.99 5.98 5.41 5.41 5.41 5.40 5.38 5.37 5.31 5.31 5.30 5.30 5.29 5.29 5.28 5.28 4.55 4.55 4.55 4.54 4.54 4.54 4.25 4.24 4.24 4.23 4.23 4.22 4.22 4.21 4.20 4.19 4.15 4.14 4.13 4.11 4.08 4.07 4.07 4.06 4.05 4.04 4.04 4.03 4.01 1.16 1.15 1.14 1.13 1.13 1.11
12
0.99
0.91
4.45
1.00
1.04
1.04
1.04
2.04
8.68
4.73
9.44
13.14
1.0 10.5 10.0 9.5 9.0 8.5 8.0 7.5 7.0 6.5 6.0 5.5 5.0 4.5 4.0 3.5 3.0 2.5 2.0 1.5 1.0 0.5
f1 (ppm)
9000 8500 8000 7500 7000 6500 6000 5500 5000 4500 4000 3500 3000 2500 2000 1500 1000 500 0 -500

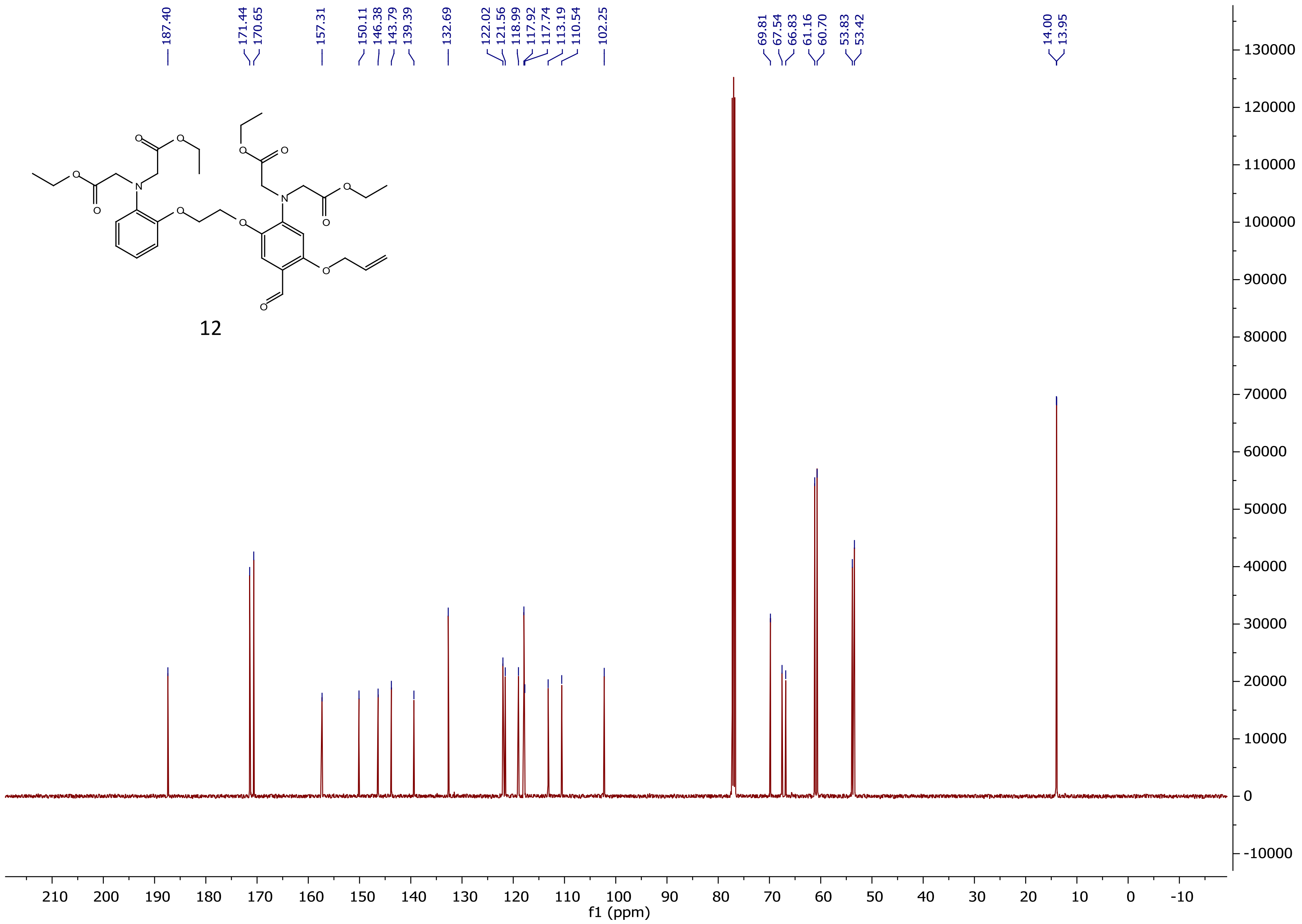
187.40
171.44
170.65
157.31
150.11
146.38
143.79
139.39
132.69
122.02
121.56
118.99
117.92
117.74
113.19
110.54
102.25
69.81
67.54
66.83
61.16
60.70
53.83
53.42
14.00
13.95
12
130000
120000
110000
100000
90000
80000
70000
60000
50000
40000
30000
20000
10000
0
-10000
210
200
190
180
170
160
150
140
130
120
110
100
90
80
70
60
50
40
30
20
10
0
-10
f1 (ppm)

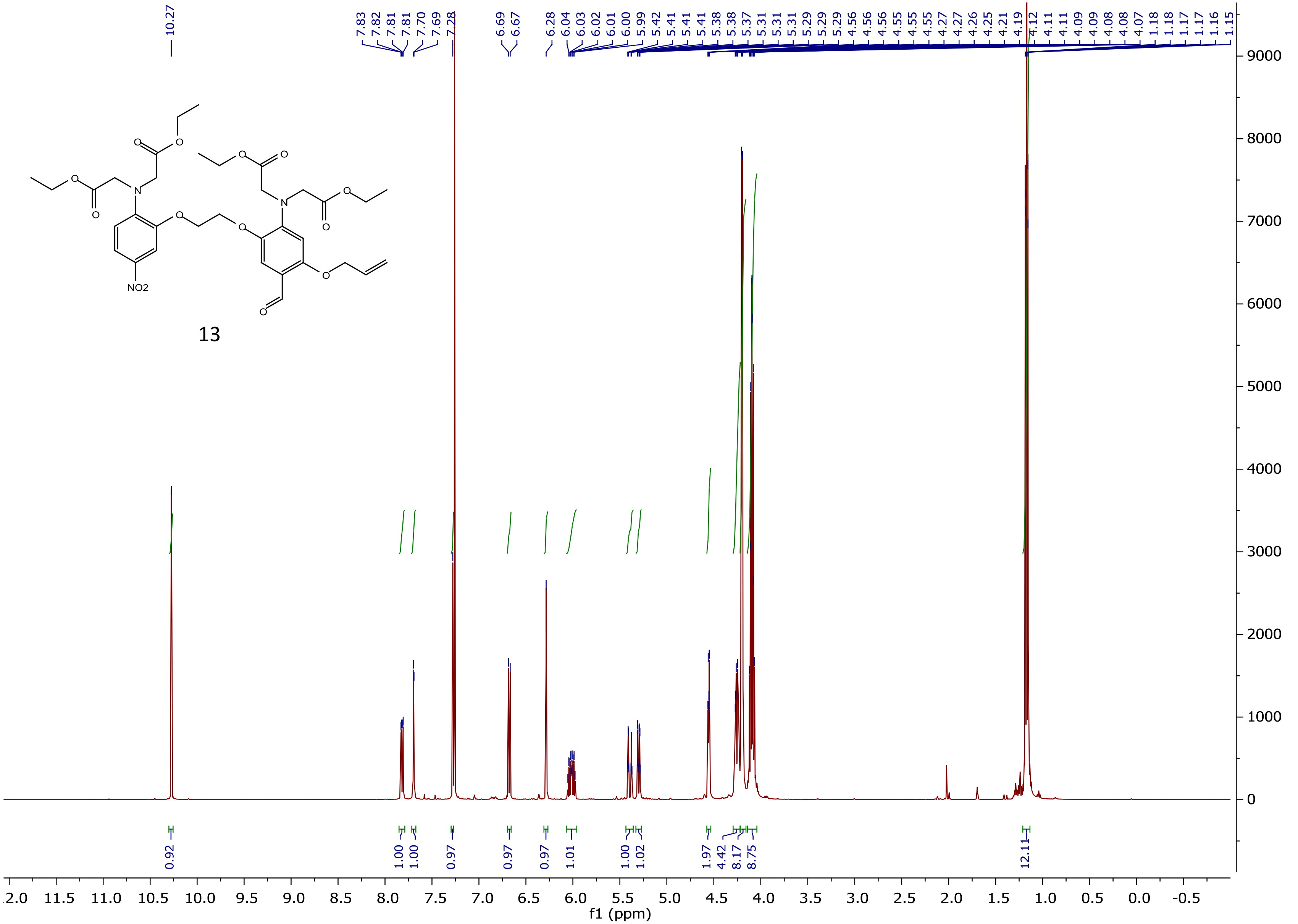

10.27
7.83 7.82 7.81 7.81 7.70 7.69 7.28
6.69 6.67
6.28 6.04 6.03 6.02 6.01 6.00 5.99 5.42 5.41 5.41 5.38 5.38 5.37 5.31 5.31 5.29 5.29 4.56 4.56 4.56 4.55 4.55 4.55 4.27 4.27 4.26 4.25 4.21 4.19 4.12 4.11 4.11 4.09 4.09 4.08 4.08 4.07 1.18 1.17 1.17 1.16 1.15
NO2
13
0.92 1.00 1.00 0.97 0.97 0.97 1.01 1.00 1.02 1.97 4.42 8.17 8.75 12.11
.2.0 11.5 11.0 10.5 10.0 9.5 9.0 8.5 8.0 7.5 7.0 6.5 6.0 5.5 5.0 4.5 4.0 3.5 3.0 2.5 2.0 1.5 1.0 0.5 0.0 -0.5
f1 (ppm)
9000 8000 7000 6000 5000 4000 3000 2000 1000 0

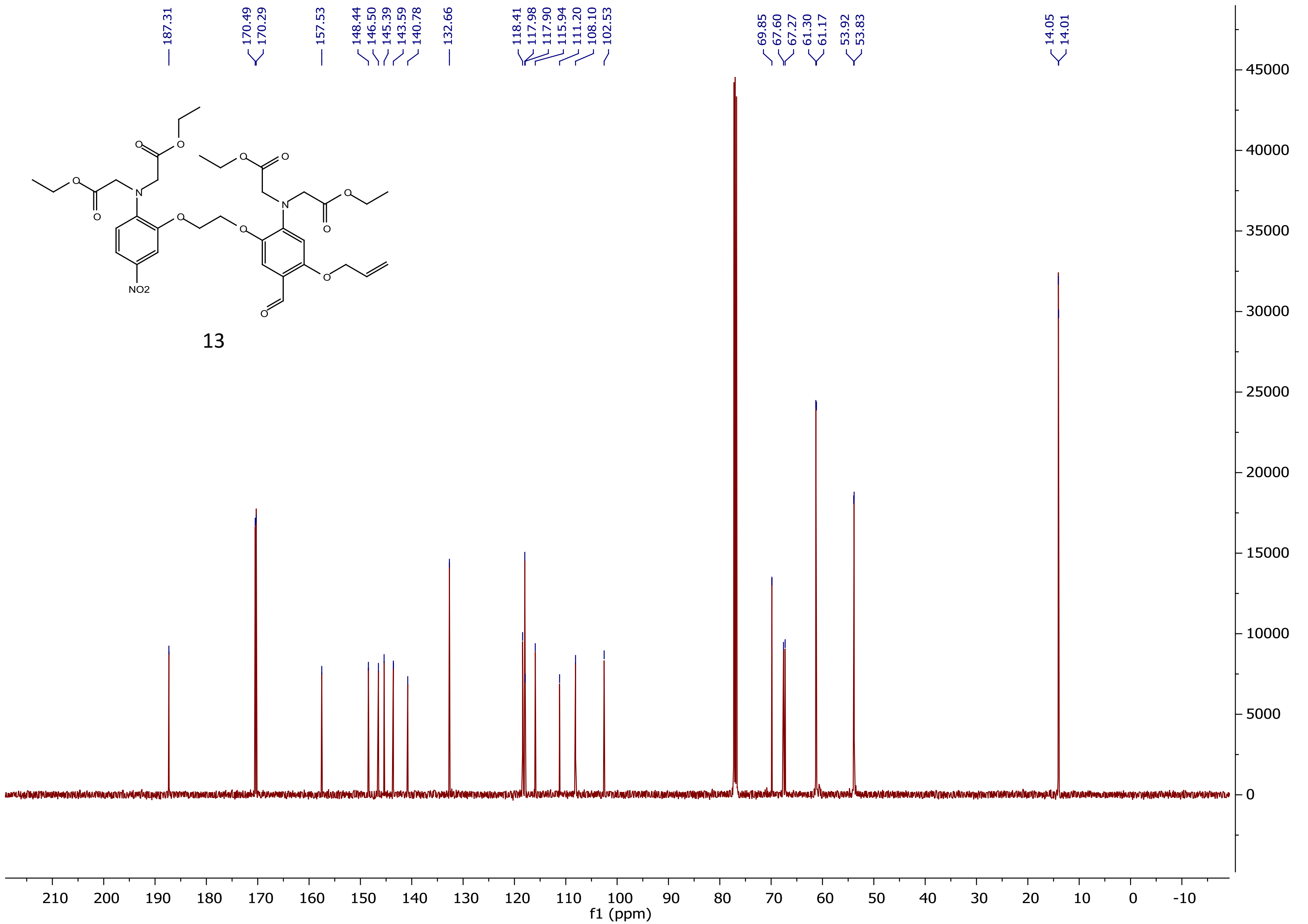
187.31
170.49
170.29
157.53
148.44
146.50
145.39
143.59
140.78
132.66
118.41
117.98
117.90
115.94
111.20
108.10
102.53
69.85
67.60
67.27
61.30
61.17
53.92
53.83
14.05
14.01
NO2
f1 (ppm)

13

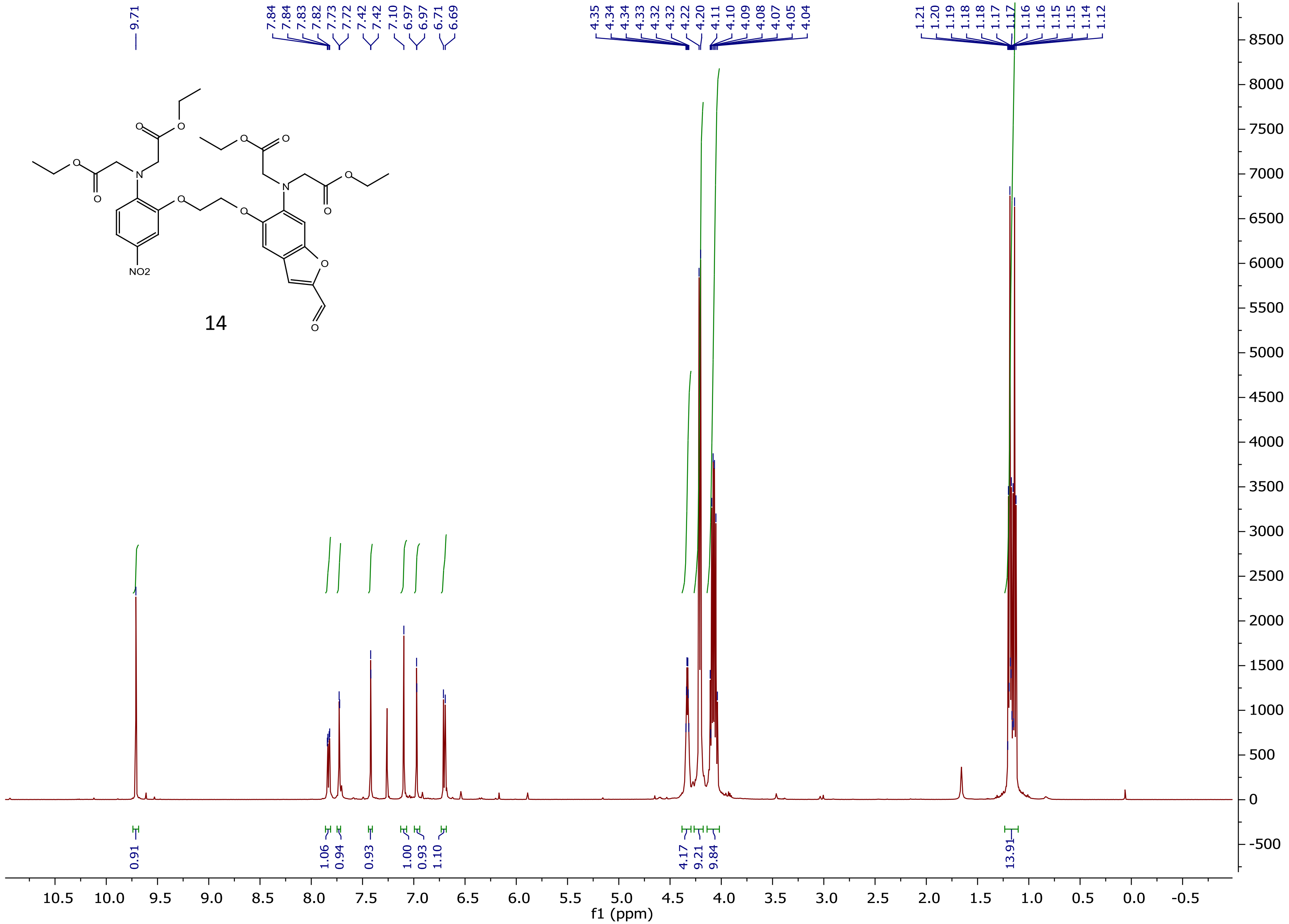
9.71
7.84
7.84
7.83
7.82
7.73
7.72
7.42
7.42
7.10
6.97
6.97
6.71
6.69
4.35
4.34
4.34
4.33
4.32
4.32
4.22
4.20
4.11
4.10
4.09
4.08
4.07
4.05
4.04
1.21
1.20
1.19
1.18
1.18
1.17
1.17
1.16
1.16
1.15
1.15
1.14
1.12
O
O
O
O
N
O
O
O
O
O
N
O
O
O
O
NO2
O
O
0.91
1.06
0.94
0.93
1.00
0.93
1.10
4.17
9.21
9.84
13.91
10.5
10.0
9.5
9.0
8.5
8.0
7.5
7.0
6.5
6.0
5.5
5.0
4.5
4.0
3.5
3.0
2.5
2.0
1.5
1.0
0.5
0.0
-0.5
f1 (ppm)
8500
8000
7500
7000
6500
6000
5500
5000
4500
4000
3500
3000
2500
2000
1500
1000
500
0
-500

14

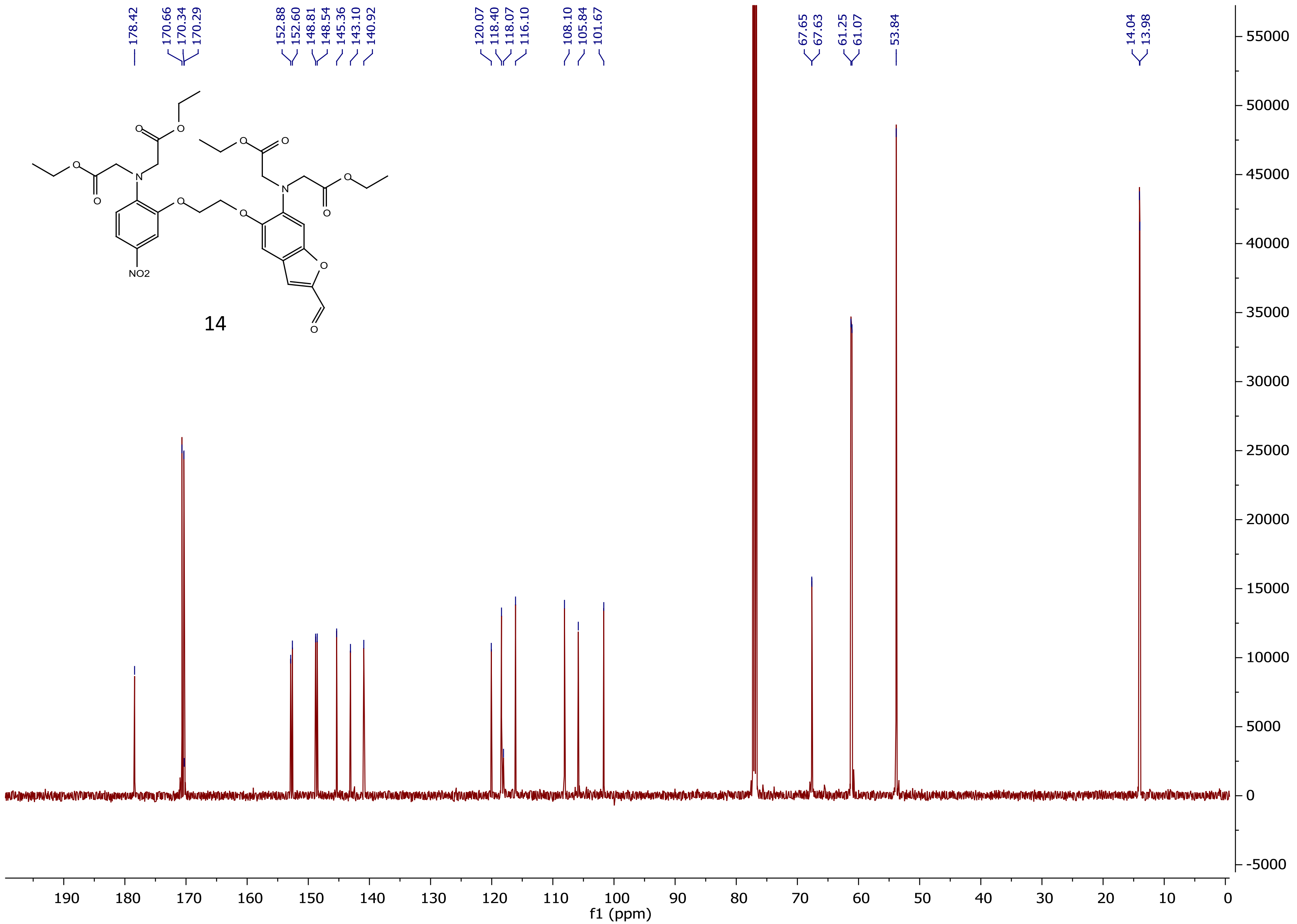

178.42
170.66
170.34
170.29
152.88
152.60
148.81
148.54
145.36
143.10
140.92
120.07
118.40
118.07
116.10
108.10
105.84
101.67
67.65
67.63
61.25
61.07
53.84
14.04
13.98
NO2
14
f1 (ppm)

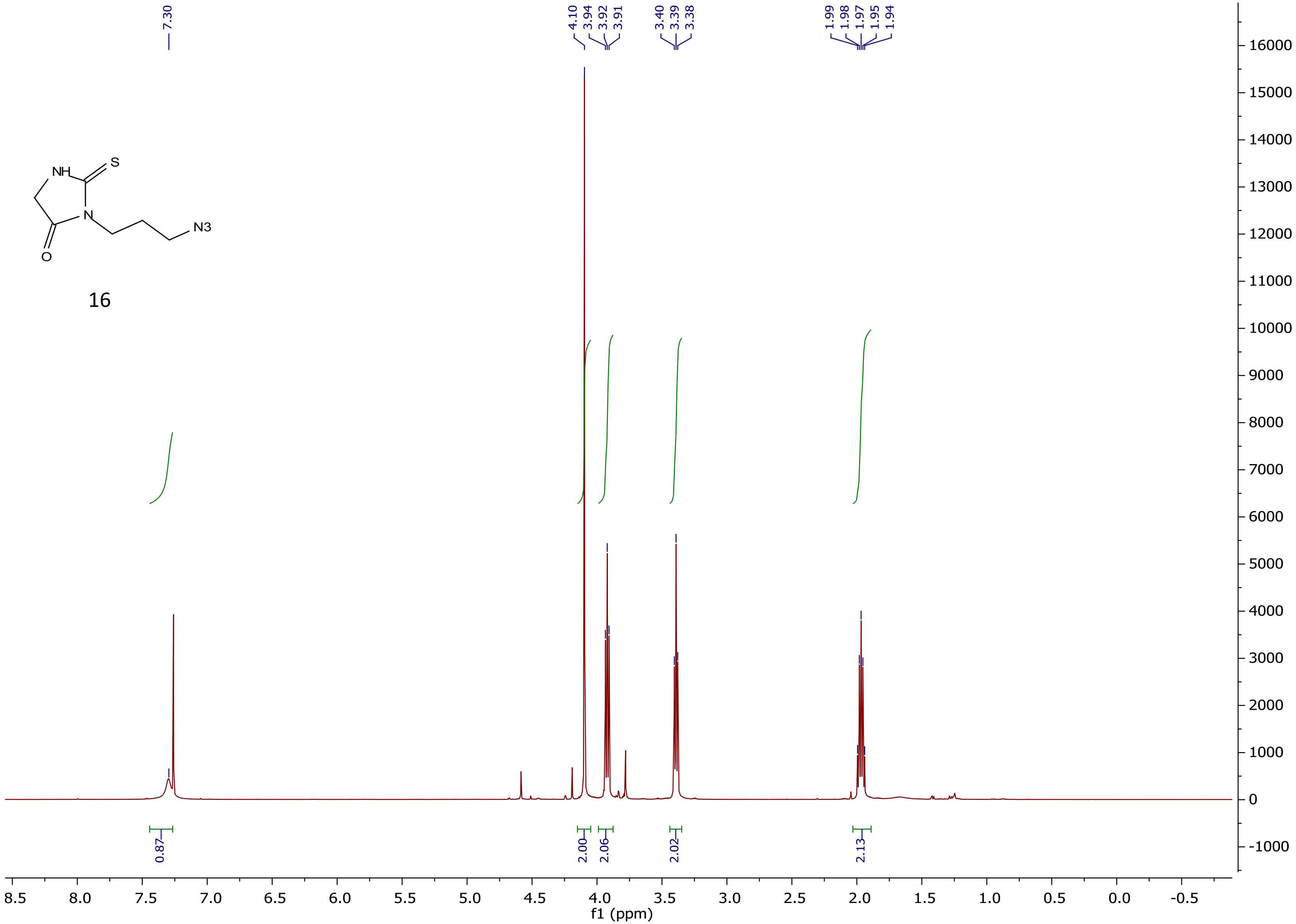
7.30
4.10
3.94
3.92
3.91
3.40
3.39
3.38
1.99
1.98
1.97
1.95
1.94
NH
S
N
N3
O
16
0.87
2.00
2.06
2.02
2.13
8.5 8.0 7.5 7.0 6.5 6.0 5.5 5.0 4.5 4.0 3.5 3.0 2.5 2.0 1.5 1.0 0.5 0.0 -0.5
f1 (ppm)
16000
15000
14000
13000
12000
11000
10000
9000
8000
7000
6000
5000
4000
3000
2000
1000
0
-1000

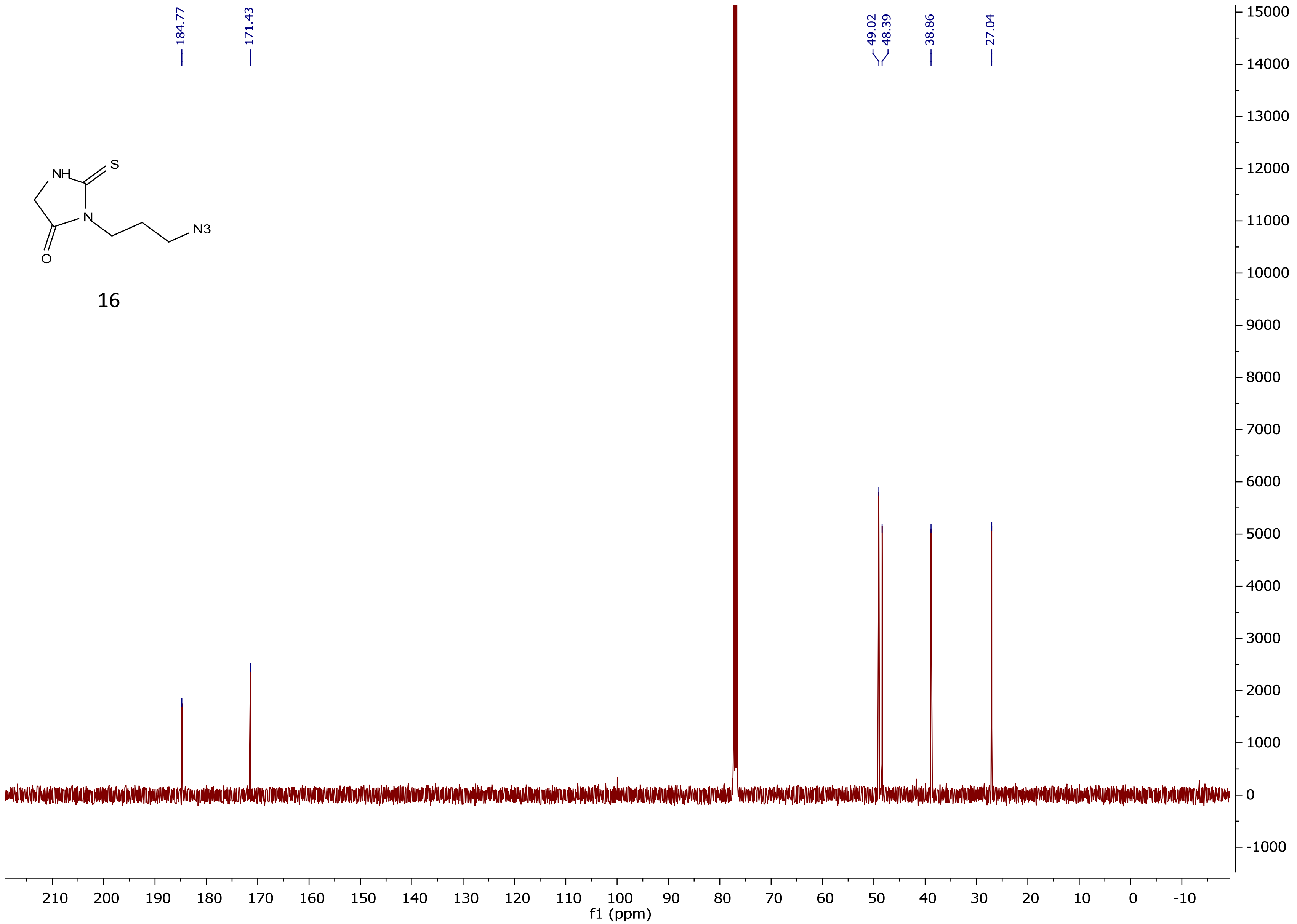

184.77
171.43
49.02
48.39
38.86
27.04
NH
S
N
N3
O
16
f1 (ppm)

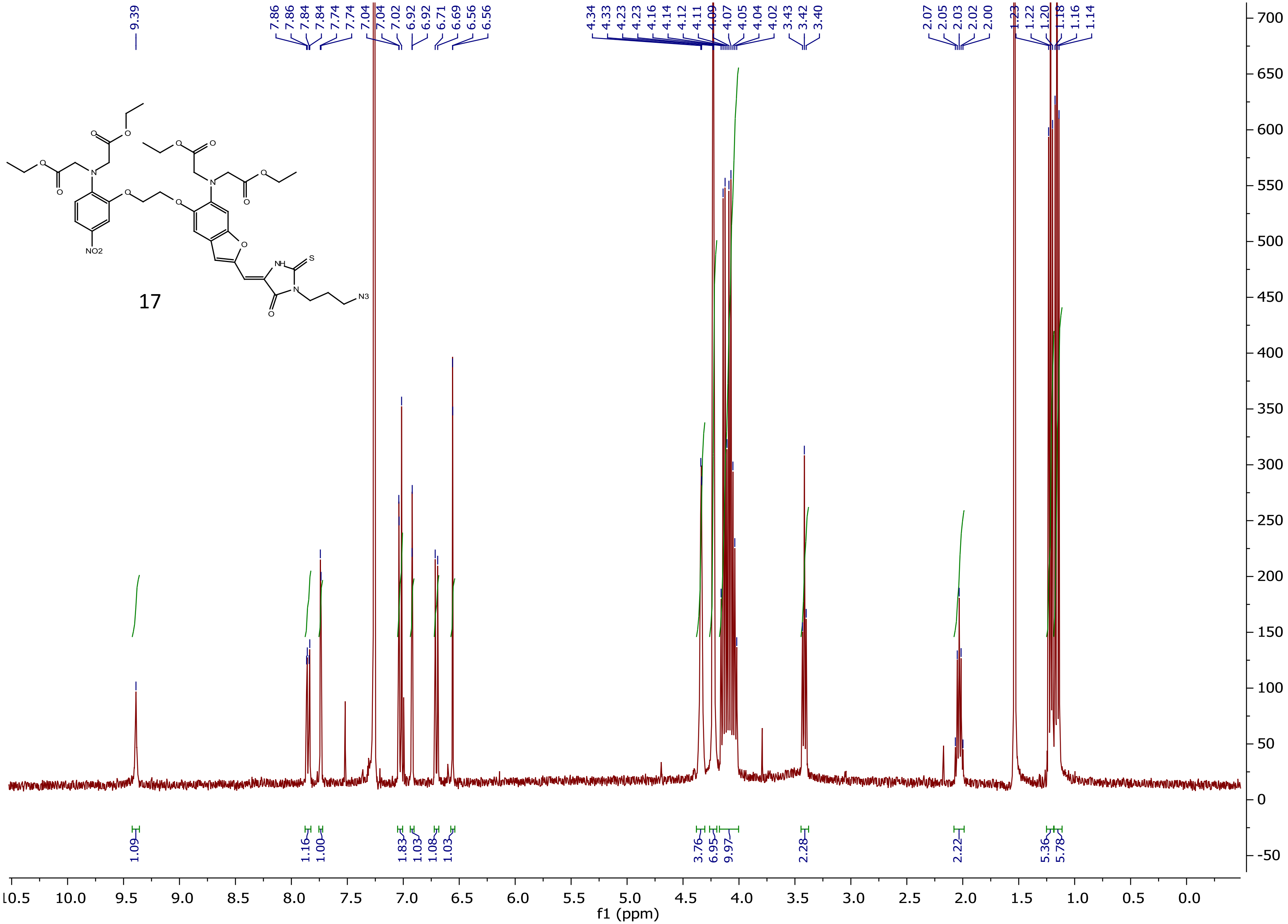
9.39
7.86 7.86 7.84 7.84 7.74 7.74 7.04 7.04 7.02 6.92 6.92 6.71 6.69 6.56 6.56
4.34 4.33 4.23 4.23 4.16 4.14 4.12 4.11 4.09 4.07 4.05 4.04 4.02 3.43 3.42 3.40
2.07 2.05 2.03 2.02 2.00 1.23 1.22 1.20 1.18 1.16 1.14
NO2
NH
S
N3
O
N
17
1.09
1.16
1.00
1.83
1.03
1.08
1.03
3.76
6.95
9.97
2.28
2.22
5.36
5.78
f1 (ppm)

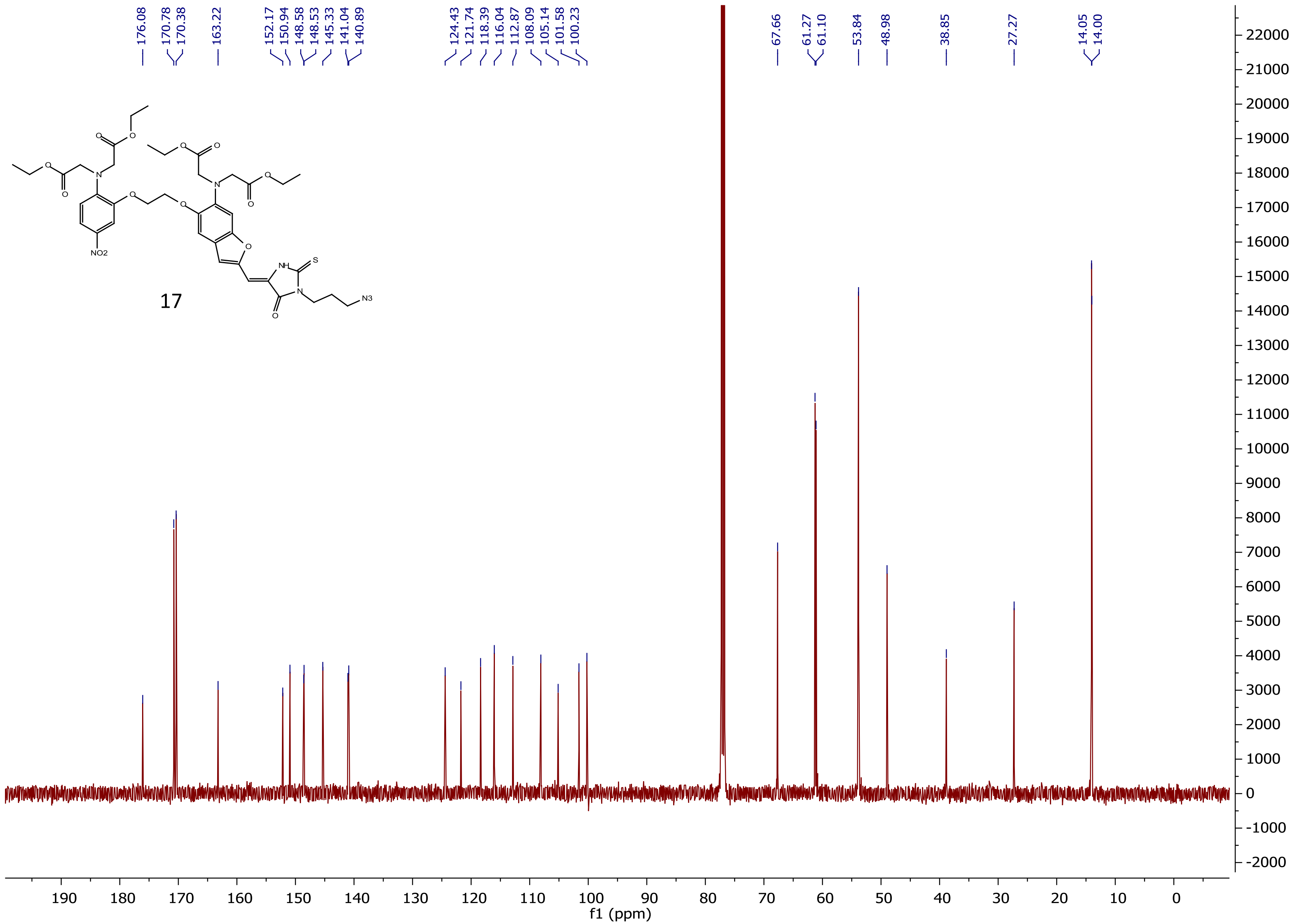
176.08
170.78
170.38
163.22
152.17
150.94
148.58
148.53
145.33
141.04
140.89
124.43
121.74
118.39
116.04
112.87
108.09
105.14
101.58
100.23
67.66
61.27
61.10
53.84
48.98
38.85
27.27
14.05
14.00
17
f1 (ppm)

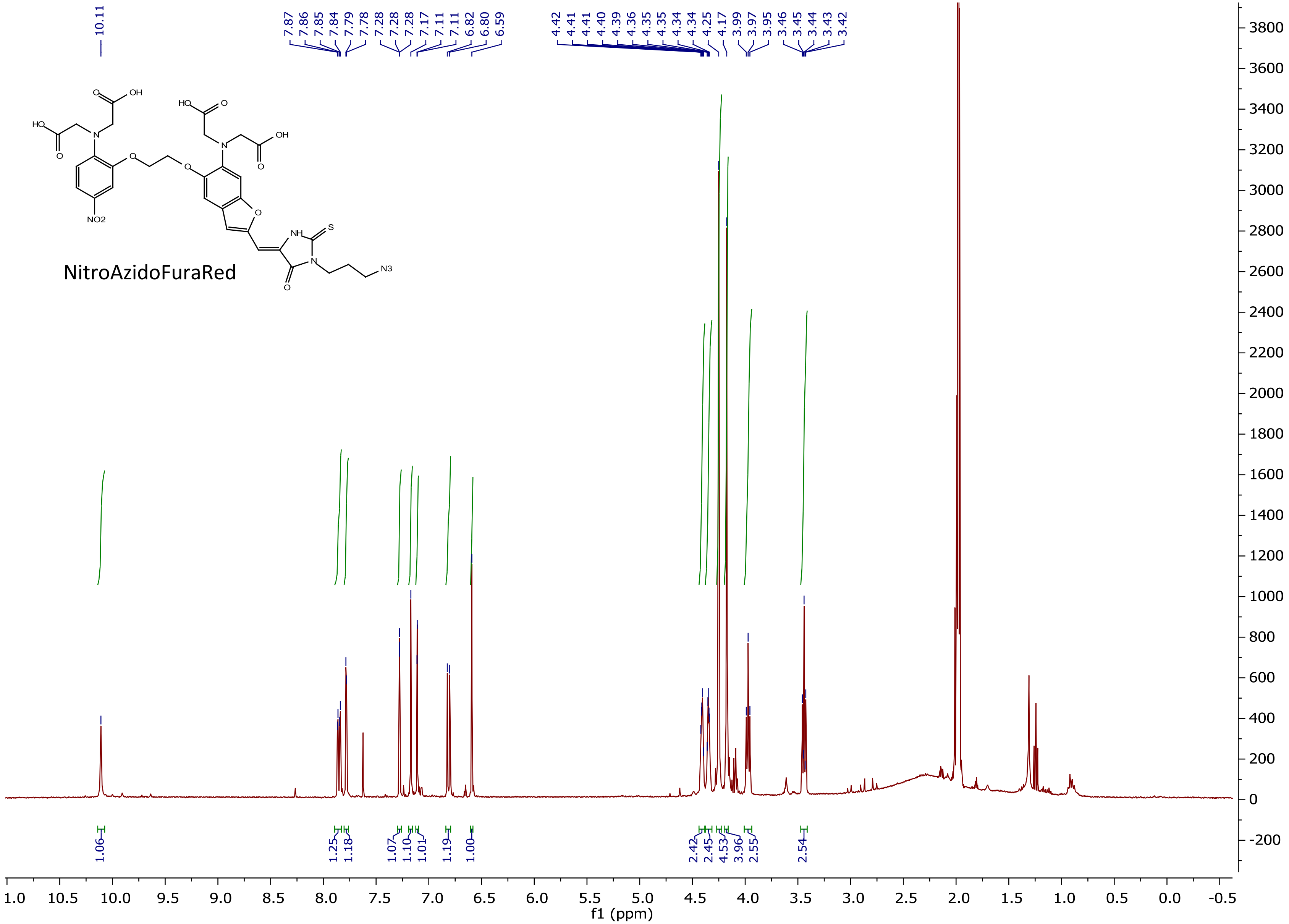
NitroAzidoFuraRed
10.11
7.87 7.86 7.85 7.84 7.79 7.78 7.28 7.28 7.28 7.17 7.11 7.11 6.82 6.80 6.59
4.42 4.41 4.41 4.40 4.39 4.36 4.35 4.35 4.34 4.34 4.25 4.17 3.99 3.97 3.95 3.46 3.45 3.44 3.43 3.42
1.06 1.25 1.18 1.07 1.10 1.01 1.19 1.00 2.42 2.45 4.53 3.96 2.55 2.54
f1 (ppm)

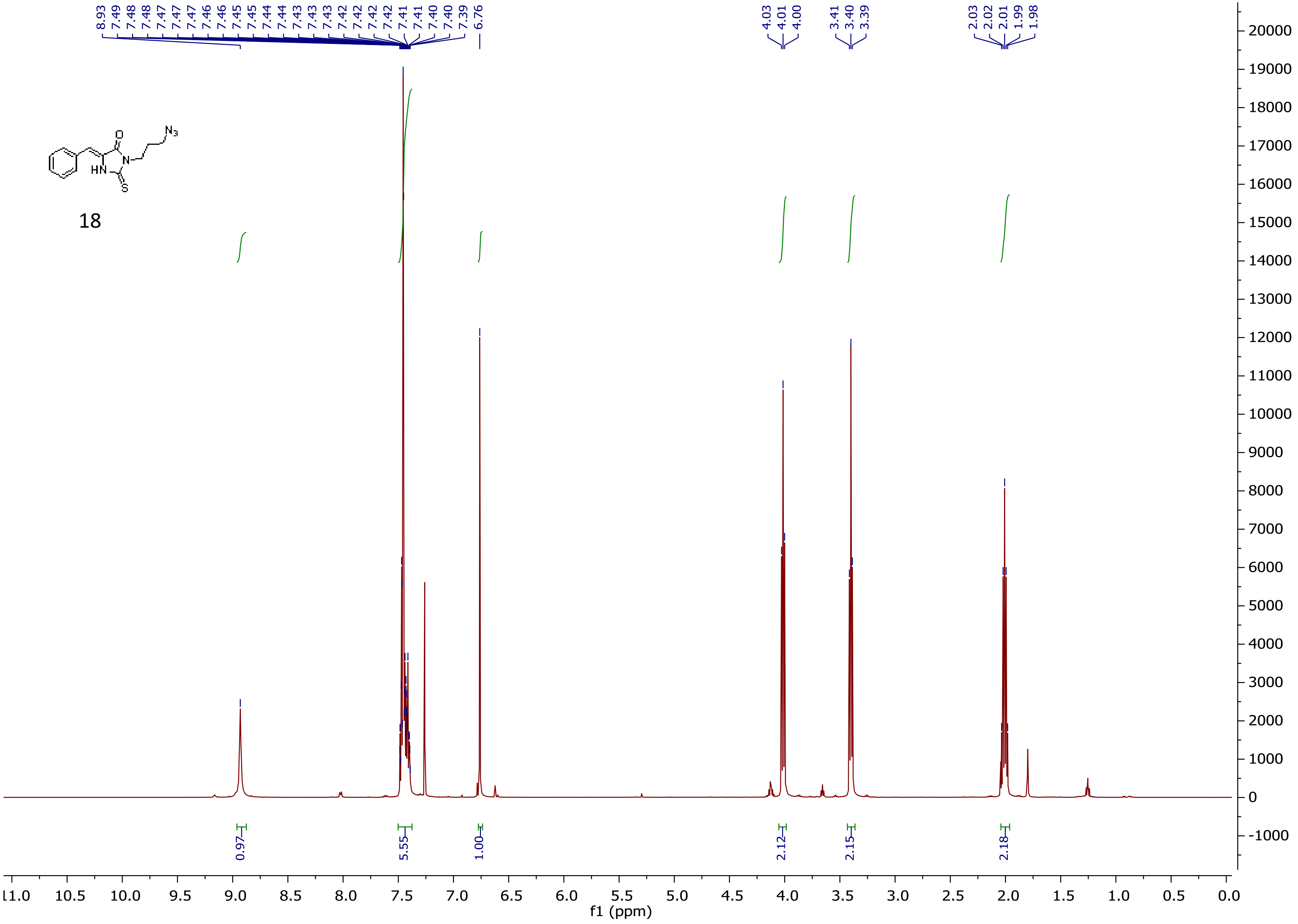
8.93 7.49 7.48 7.48 7.47 7.47 7.47 7.46 7.46 7.45 7.45 7.44 7.44 7.43 7.43 7.43 7.42 7.42 7.42 7.42 7.41 7.41 7.40 7.40 7.39 6.76
4.03 4.01 4.00
3.41 3.40 3.39
2.03 2.02 2.01 1.99 1.98
N3
O
N
HN
S
18
0.97
5.55
1.00
2.12
2.15
2.18
f1 (ppm)

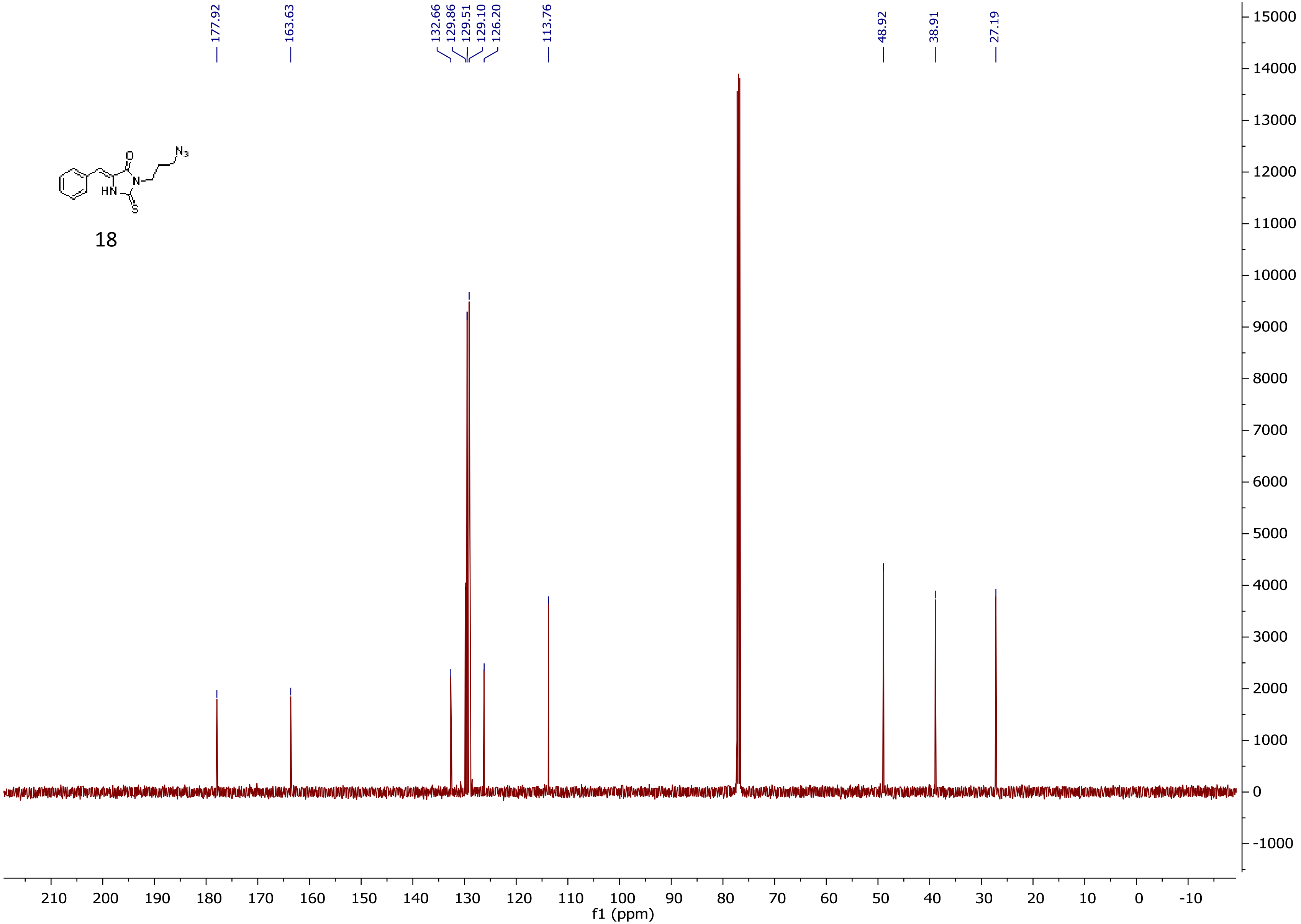
O
N_3
N
HN
S
177.92
163.63
132.66
129.86
129.51
129.10
126.20
113.76
48.92
38.91
27.19
15000
14000
13000
12000
11000
10000
9000
8000
7000
6000
5000
4000
3000
2000
1000
0
-1000
210 200 190 180 170 160 150 140 130 120 110 100 90 80 70 60 50 40 30 20 10 0 -10
f1 (ppm)

18

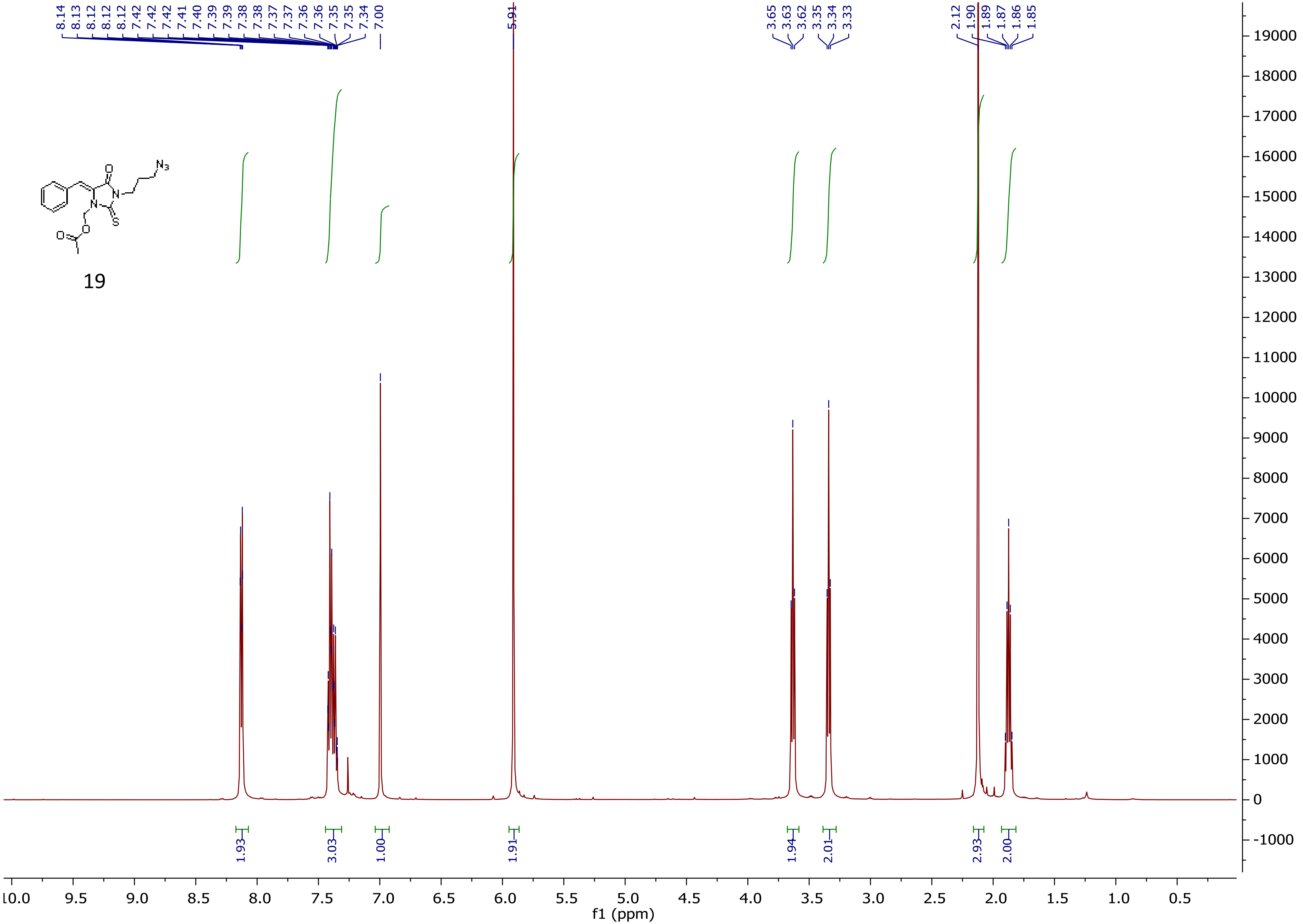
8.14 8.13 8.12 8.12 8.12 7.42 7.42 7.42 7.41 7.40 7.39 7.39 7.38 7.38 7.37 7.37 7.36 7.36 7.35 7.35 7.34 7.00
5.91
3.65 3.63 3.62 3.35 3.34 3.33
2.12 1.90 1.89 1.87 1.86 1.85
19
1.93 3.03 1.00 1.91 1.94 2.01 2.93 2.00
f1 (ppm)

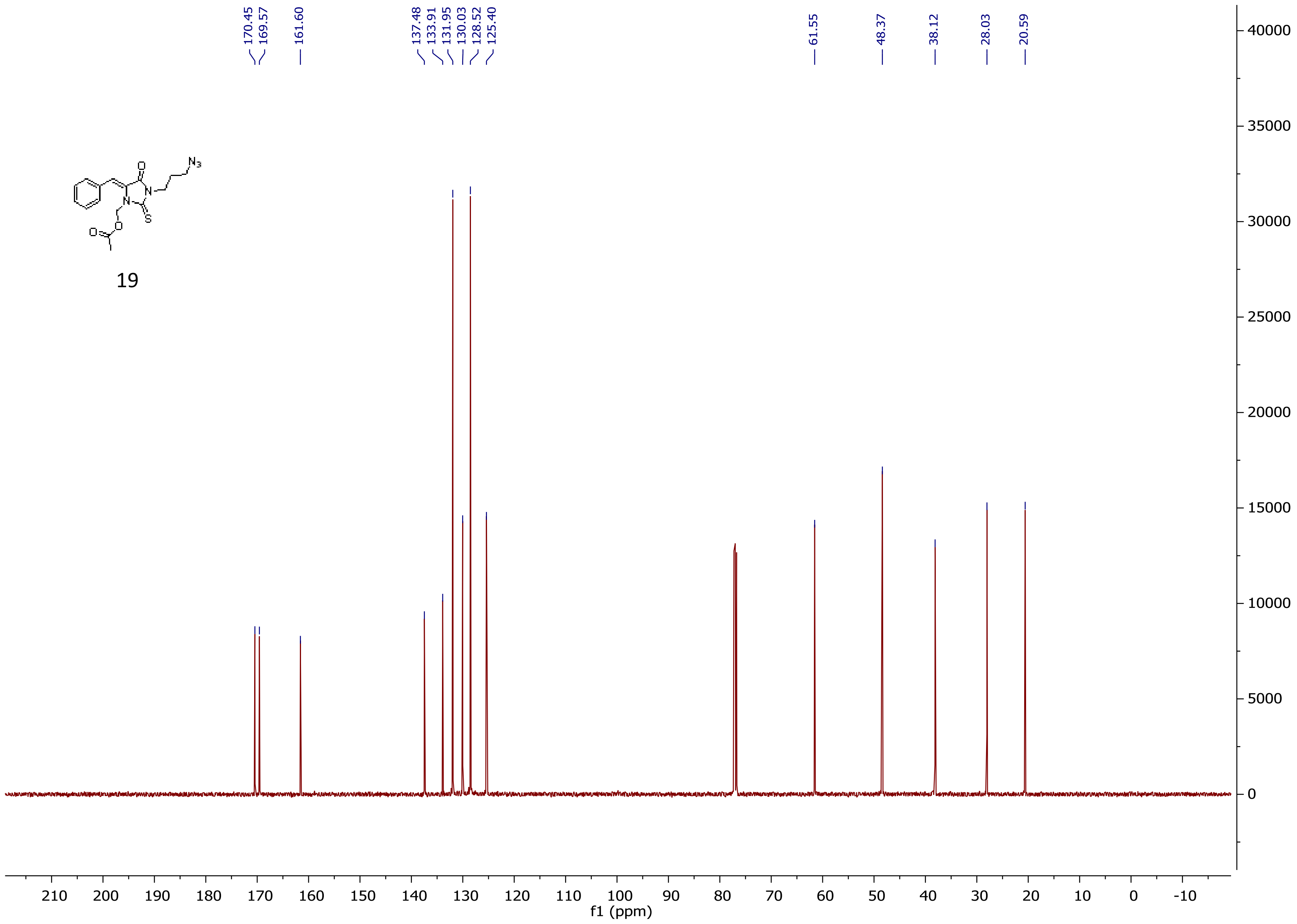
170.45
169.57
161.60
137.48
133.91
131.95
130.03
128.52
125.40
61.55
48.37
38.12
28.03
20.59
40000
35000
30000
25000
20000
15000
10000
5000
0
210
200
190
180
170
160
150
140
130
120
110
100
90
80
70
60
50
40
30
20
10
0
-10
f1 (ppm)
N3
O
N
N
S
O
O

19

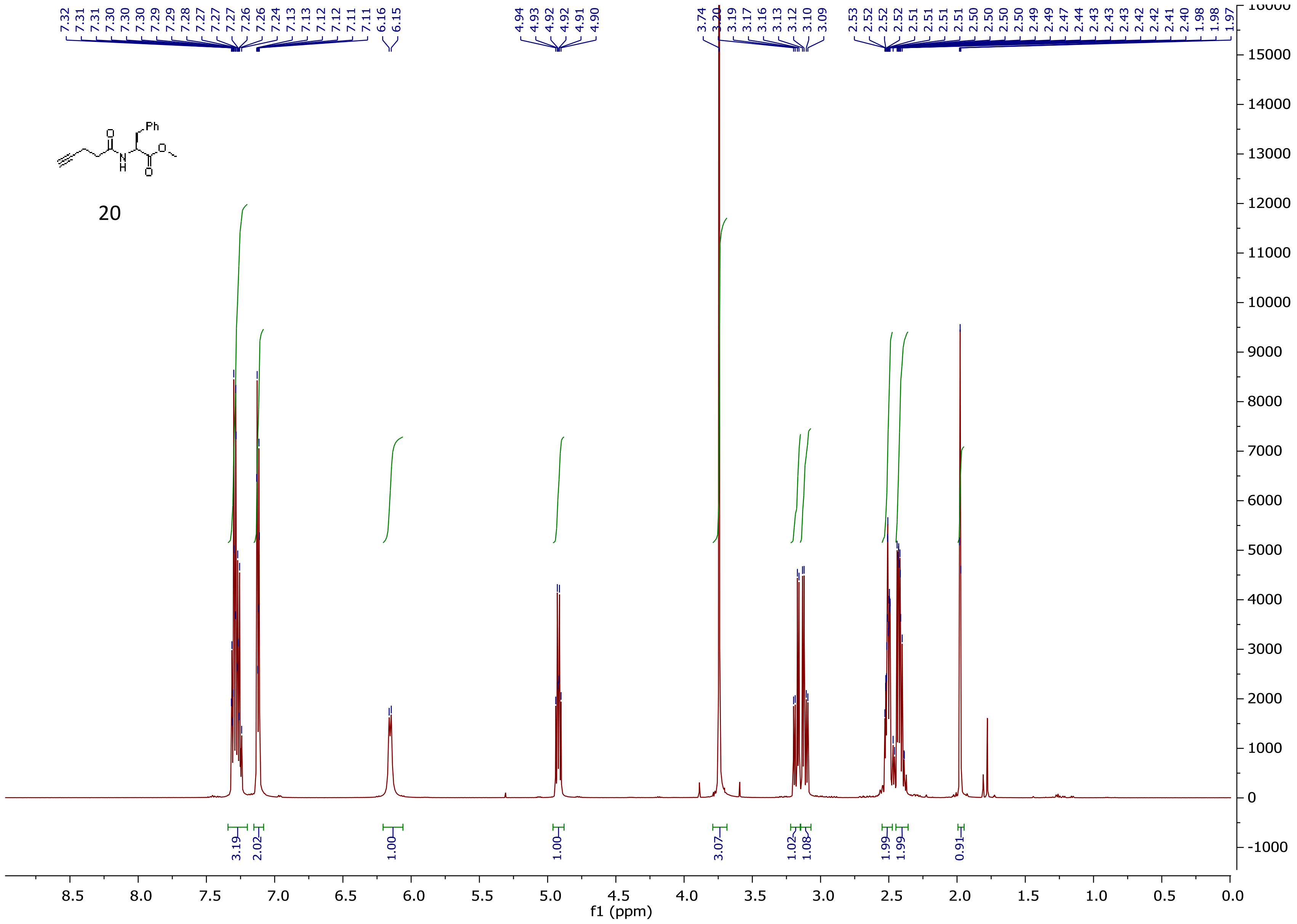

20

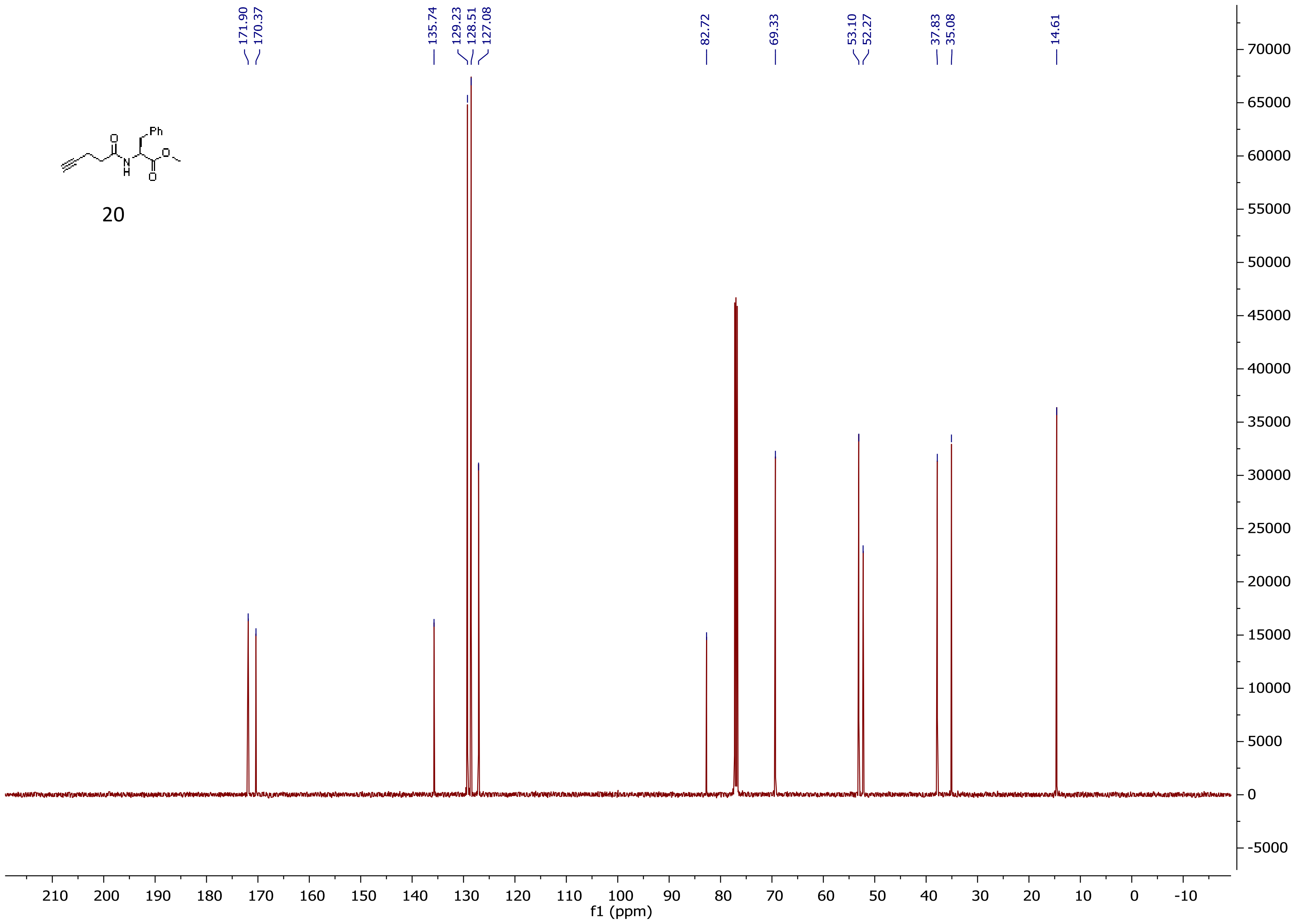
171.90
170.37
135.74
129.23
128.51
127.08
82.72
69.33
53.10
52.27
37.83
35.08
14.61
Ph
O
N
H
O
O
70000
65000
60000
55000
50000
45000
40000
35000
30000
25000
20000
15000
10000
5000
0
-5000
210 200 190 180 170 160 150 140 130 120 110 100 90 80 70 60 50 40 30 20 10 0 -10
f1 (ppm)

20

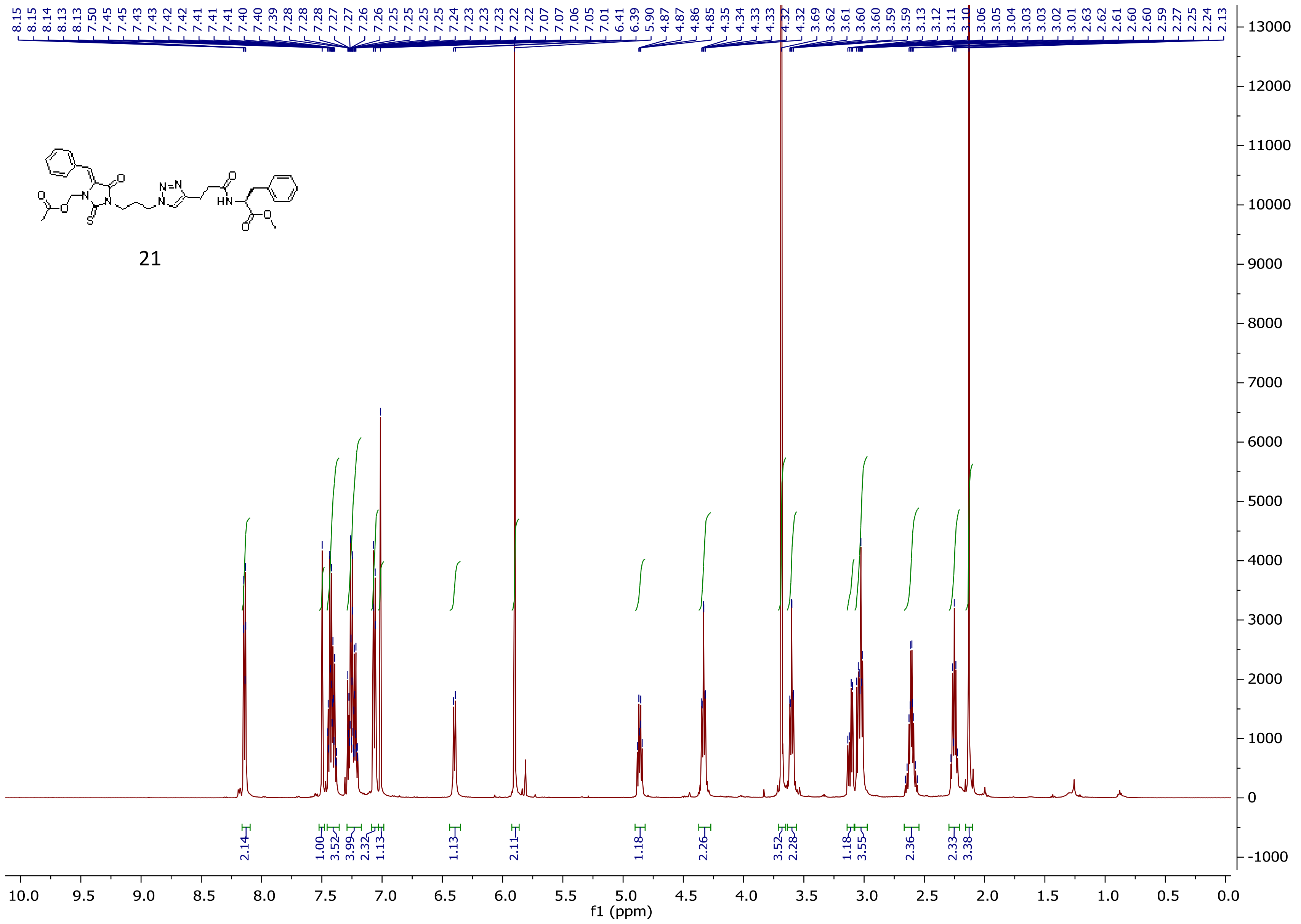
21
f1 (ppm)

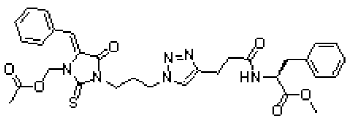

21

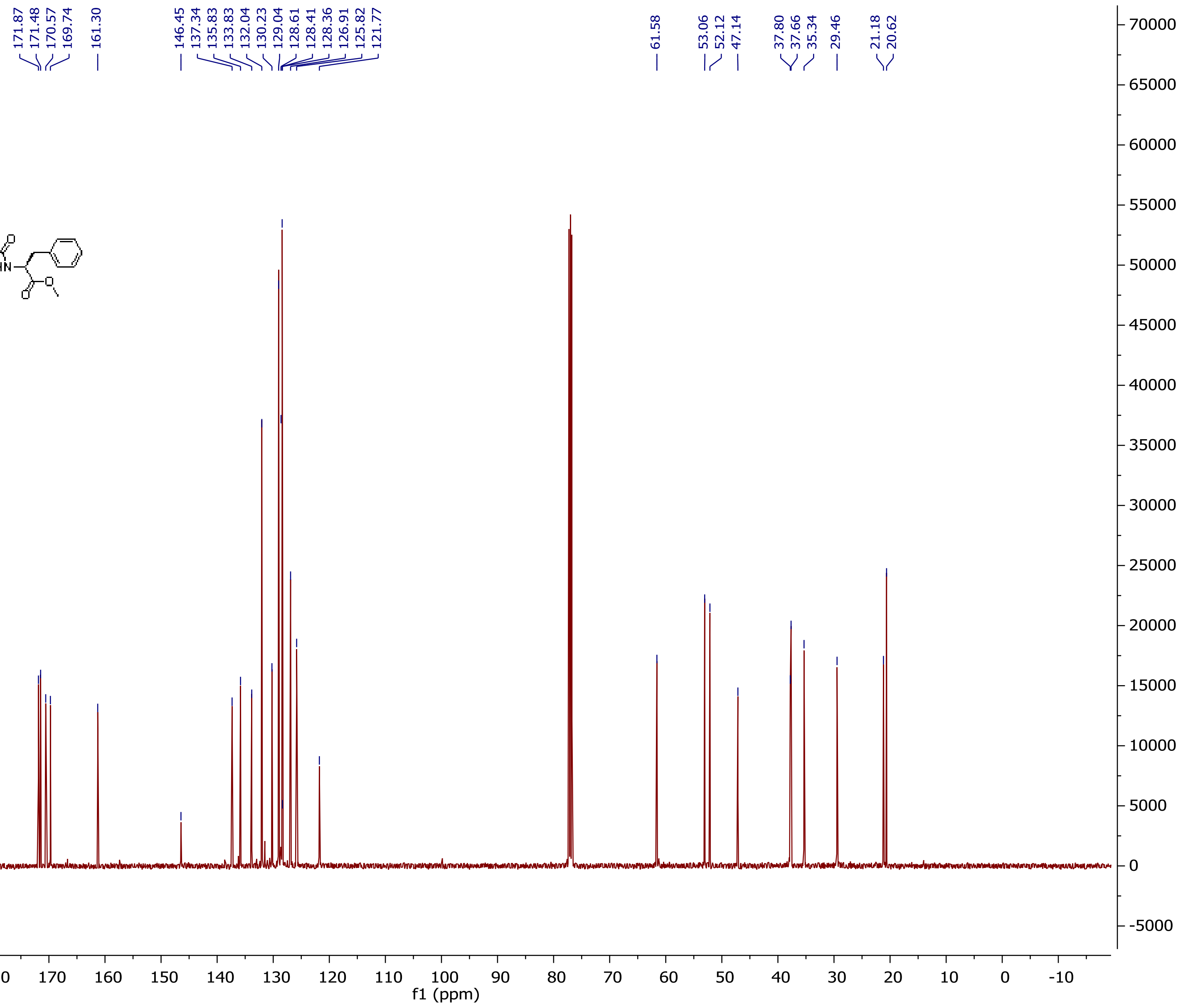

171.87
171.48
170.57
169.74
161.30
146.45
137.34
135.83
133.83
132.04
130.23
129.04
128.61
128.41
128.36
126.91
125.82
121.77
61.58
53.06
52.12
47.14
37.80
37.66
35.34
29.46
21.18
20.62
f1 (ppm)
